# Progressive microbial adaptation of the bovine rumen and hindgut in response to a step-wise increase in dietary starch and the influence of phytogenic supplementation

**DOI:** 10.3389/fmicb.2022.920427

**Published:** 2022-07-22

**Authors:** Sara Ricci, Cátia Pacífico, Ezequias Castillo-Lopez, Raul Rivera-Chacon, Heidi E. Schwartz-Zimmermann, Nicole Reisinger, Franz Berthiller, Qendrim Zebeli, Renee M. Petri

**Affiliations:** ^1^Christian Doppler Laboratory for Innovative Gut Health Concepts of Livestock, Department for Farm Animals and Veterinary Public Health, Institute of Animal Nutrition and Functional Plant Compounds, University of Veterinary Medicine, Vienna, Austria; ^2^Christian Doppler Laboratory for Innovative Gut Health Concepts of Livestock, Department of Agrobiotechnology (IFA-Tulln), Institute of Bioanalytics and Agro-Metabolomics, University of Natural Resources and Life Sciences, Vienna, Austria; ^3^DSM, BIOMIN Research Center, Tulln, Austria; ^4^Agriculture and Agri-Food Canada, Sherbrooke Research and Development Centre, Sherbrooke, QC, Canada

**Keywords:** cattle, feces, microbial activity, concentrate diet, grain, phytogenic additive, metabolomics, microbiota

## Abstract

Microbial composition and activity in the gastrointestinal tract (GIT) of cattle has important implications for animal health and welfare, driving the focus of research toward ways to modify their function and abundance. However, our understanding of microbial adaption to nutritional changes remains limited. The aim of this study was to examine the progressive mechanisms of adaptation in the rumen and hindgut of cattle receiving increasing amounts of starch with or without dietary supplementation of a blended phytogenic feed additive (PFA; containing menthol, thymol and eugenol). We used 16S rRNA gene amplicon sequencing to assess the microbial composition and predicted metabolic pathways in ruminal solid and liquid digesta, and feces. Furthermore, we employed targeted liquid chromatography-mass spectrometry methods to evaluate rumen fluid metabolites. Results indicated a rapid microbial adaptation to diet change, starting on the second day of starch feeding for the particle associated rumen liquid (PARL) microbes. Solid rumen digesta- and feces-associated microbes started changing from the following day. The PARL niche was the most responsive to dietary changes, with the highest number of taxa and predicted pathways affected by the increase in starch intake, as well as by the phytogenic supplementation. Despite the differences in the microbial composition and metabolic potential of the different GIT niches, all showed similar changes toward carbohydrate metabolism. Metabolite measurement confirmed the high prevalence of glucose and volatile fatty acids (VFAs) in the rumen due to the increased substrate availability and metabolic activity of the microbiota. Families *Prevotellaceae, Ruminococcaceae* and *Lachnospiraceae* were found to be positively correlated with carbohydrate metabolism, with the latter two showing wide-ranging predicted metabolic capabilities. Phytogenic supplementation affected low abundant taxa and demonstrated the potential to prevent unwanted implications of feeding high-concentrate diet, such as reduction of microbial diversity. The inclusion of 50% concentrate in the diet caused a major shift in microbial composition and activity in the GIT of cattle. This study demonstrated the ability of microorganisms in various GIT niches to adjust differentially, yet rapidly, to changing dietary conditions, and revealed the potential beneficial effects of supplementation with a PFA during dietary adaptation.

## Introduction

The importance of microbiota composition and activity, in terms of the complex mechanisms regulating animal health, metabolic function and production, has come into focus in recent years (O'Hara et al., 2020). Among the many different factors that can affect microbiota structure and function, including genetics, environment, and metabolic state, diet remains the key driver of change (Deusch et al., 2017; De Angelis et al., 2020). In dairy cattle, diet composition is critical for ensuring that high energy requirements for milk production are met (Zebeli et al., 2008). Research has focused on increasing the efficiency of feed utilization, avoiding severe negative energy balance, improving animal health and welfare, and reducing methane emissions, all through modulation of the rumen and hindgut microbiota (Humer et al., 2018b; Petri et al., 2018; Matthews et al., 2019). Changes in dietary composition, especially during the period of adaptation, pose an increased risk for dysbiosis of the gut microbiota (David et al., 2014). When a microbial niche is subject to an external perturbation, such as an oversupply of substrates, there is a system of adaptations in metabolic activity driving changes in ecological composition until a new level of homeostasis is reached. In gastrointestinal microbial ecosystems, this new steady state can be detrimental for the host resulting in inflammation and illness (Sommer et al., 2017; Lachnit et al., 2019).

Compared with other carbohydrate sources in dairy diets, starch is rapidly degraded by ruminal bacteria into glucose, which is transformed into pyruvate and then into end-products essential for the animals, such as acetate, propionate, and butyrate (Hoover and Miller, 1991; Mills et al., 1999; Tester et al., 2004). Supplying increased amounts of starch provides the cow with the additional energy required for milk production. However, an excessive amount of starch causes an accumulation of organic acids in the rumen due to a lack of buffering mechanisms, which can affect both the stability of the gut microbiota and animal health (Aschenbach et al., 2011). In fact, under lower pH values, some bacterial species thrive to the detriment of others, and the metabolic profile changes as a result (Russell and Rychlik, 2001; Ametaj et al., 2010). Such conditions can cause the proliferation of pathogens, as well as the production of harmful compounds (Kleen et al., 2003; Plaizier et al., 2012). The resulting dysbiotic status, if prolonged, can cause subsequent health issues, such as subacute ruminal acidosis (SARA) and inflammation in cattle (Plaizier et al., 2008; Khafipour et al., 2016). Previous research has demonstrated the capacity of microbiota to adapt and change in response to dietary variations (Petri et al., 2013; Wetzels et al., 2016), as well as their resilience potential (Weimer, 2015). Nevertheless, there is still a lack of understanding with regard to the progressive adaptation mechanisms of the GIT microbiota to increases in dietary starch. Furthermore, studies employing phytogenic feed additives (PFA) as supplement to high concentrate feeding have successfully prevented significant shifts in microbiota composition and reduced the production of harmful metabolites (Cardozo et al., 2006; Neubauer et al., 2018). While these phytogenic compounds can result in immediate changes to rumen fermentation, with the potential to alter the hindgut as well, and to help in the recovery of a perturbed ecosystem, the mechanisms of action remain largely unknown (Neubauer et al., 2018; Zhou et al., 2019).

The aim of our study was to increase the understanding of how a rapid shift in substrates affects the microbiota inhabiting different GIT niches in dairy cattle, by investigating the daily adaptation of the microorganisms, as well as their predicted metabolism and metabolite production, to increase amounts of readily fermentable carbohydrates introduced with the diet. Furthermore, we aimed to examine if supplementation with a blended PFA would mitigate and prevent a possible dysbiosis, by modulating the proliferation and activity of the ruminal and fecal microbiota.

## Materials and methods

### Experiment design and animal housing

The trial was conducted as part of a larger experiment at the research farm of the University of Veterinary Medicine, Vienna. The main experimental design and diets have been described in detail by Rivera-Chacon et al. (2022). In brief, the trial was a crossover design with two runs, separated by a 4-week washout period. Nine Holstein non-lactating rumen-cannulated cows (mean body weight: 992 ± 73 kg, mean age: 10.0 ± 0.8 years) were divided into two groups (control—CON and treatment—PHY). The groups were balanced for body weight, with five cows assigned to the PHY and four to the CON group in the first run. The treatments were inverted in the second run (four cows PHY and five cows CON). Animals were group-housed, and each cow had access to an individual feed bunk (through computer-regulated access gates). A period of adaptation to the grouping, feeders and basal diet occurred for 1 week before the trial started. The feeding protocol of the adaptation consisted in a step-wise replacement of the forage proportion (75% grass silage, 15% corn silage, and 10% grass hay in dry matter basis) with concentrate, starting with 10% concentrate mixture on day 1 and reaching 60% on day 6 (dry matter basis). The concentrate mixture (30.22% barley, 18.1% triticale, 23.08% bakery by-products, 24.0% rapeseed meal, 3.0% molasses, 0.8% mineral-vitamin premix for dairy cattle, 0.5% limestone, and 0.3% salt) was the same for the two groups, with the treatment group receiving a blended phytogenic feed additive at 400 mg/kg (dry matter basis) (Digestarom^®^, a mixture of herbs and spices that contains menthol, thymol and eugenol, DSM Austria GmbH). To maintain a stable intake of PHY additive throughout the trial, from day 1 to day 6, the corresponding quantities of PFA were supplemented directly into the rumen through the cannula. Diet was offered as a total mixed ration and provided once daily by an automatic feeding system (Trioliet Triomatic T15, Oldenzaal, The Netherlands), and was available *ad libitum*, along with water and mineral blocks. Orts were removed and fresh feed was delivered every morning. Daily feed intake was recorded automatically (Insentec B.V., Marknesse, The Netherlands). Starch intake was calculated based on the dry matter intake (DMI), and the chemical composition of feed ingredients was analyzed at the start and end of dietary adaptation.

### Ruminal pH measurements and conventional microbial fermentation products

Ruminal pH was measured every 15 mins with the Lethbridge Research Centre Ruminal pH Measurement System (LRCpH; Dascor Inc., CA, USA) (Penner et al., 2006). Specific calculations, as well as conversion of measured millivolts to pH, were similar to Castillo-Lopez et al. (2014b), but a pH threshold of 5.8 was used to assess ruminal acidification. All samples were collected daily, 4 h after morning feeding. Samples of ruminal fluid for VFA, ammonia and lactate analyses were collected from the ventral sac of the rumen using a 20-ml sterile syringe and stored at −20°C. Composition of VFAs was measured using gas chromatography (GC-2010 PLUS, Shimadzu) applying the protocol described by Castillo-Lopez et al. (2021). The concentration of ammonia was measured using an indophenol colorimetric method on a U3000 spectrophotometer (INULA GmbH, Vienna, Austria) (Weatherburn, 1967). Thawed samples were centrifuged at 15,115 × *g* for 10 min. Ammonia and phenol were oxidized by sodium hydroxide in the presence of sodium nitroprusside and dichloroisocyanuric acid. Absorbance was measured at 655 nm after 90 min of reaction. D-Lactate was measured with the Megazyme K-DATE assay (Megazyme Int., Ireland), according to the manufacturer's instructions.

### Ruminal metabolomics

Samples of rumen fluid for metabolomics analyses were collected 4 h after the morning feeding from the ventral sac of the rumen, snap frozen in liquid nitrogen and stored at −80°C. Metabolites were determined with anion-exchange chromatography-high resolution mass spectrometry (IC-HR-MS) and high-performance liquid chromatography coupled to tandem mass spectrometry (LC-MS/MS). Carboxylic acids, sugar phosphates, and sugars were analyzed by anion exchange chromatography on a Dionex Integrion HPIC system (Thermo Scientific) coupled to a Thermo Scientific Q Exactive Orbitrap mass spectrometer. Sample preparation consisted of shaking 20 μl of rumen fluid with 980 μl of acetonitrile/water (80:20, v/v) at 4°C for 10 min, centrifugation at 14,350 × *g* for 10 min and tenfold dilution of the supernatants with acetonitrile/water (20:80, v/v).

Biogenic amines were determined by LC-MS/MS after derivatization with phenyl isothiocyanate (PITC). Sample preparation was performed in 96-well plates using a modified protocol based on Biocrates' MxP® Quant 500 kit (Innsbruck, Austria). Briefly, a 10 μl aliquot of rumen fluid sample or different volumes of calibration stock solutions containing between 0.009 and 9 mg/L of all analytes and 30 μl of internal standard solution containing 10 mg/L ^13^C-putrescine in acetonitrile:water (50:50, v/v) were pipetted into a 96-well plate and evaporated to dryness under a stream of nitrogen. Subsequently, 50 μl of derivatization reagent (ethanol:water:pyridine:PITC 31.7:31.7:31.7:5.0, v/v/v/v) was added and the plate was covered, shaken for 20 s, and placed in the dark at room temperature for derivatization of amines. After 1 h of derivatization, the derivatization reagent was evaporated under nitrogen. Finally, analytes were extracted by shaking in 300 μl of methanol containing 4.9 mM ammonium acetate for 30 min and the extracts were centrifuged. One aliquot of the extract was used directly for LC-MS/MS measurement, while another aliquot was diluted at 1:25 with methanol prior to measurement because of substantial concentration differences of the analytes in the rumen fluid.

The chromatographic and mass spectrometric conditions as well as the quantification approaches and the used solvents and reagents are described in detail in the supplementary data in Supplementary Material. Selected reaction monitoring (SRM) for LC-MS/MS analysis is given in Supplementary Table S1.

### Microbiota analyses

#### Sample collection

Samples for microbiota analyses were collected aseptically 4 h after morning feeding, following an approach similar to Castillo-Lopez et al. (2014a). To collect samples of solid digesta and particle associated rumen liquid (PARL) (Tafaj et al., 2004), a handful of digesta was sampled from four different locations (dorsal, cranial and caudal mat and ventral sac). PARL samples were collected in a beaker by squeezing the digesta sample through four layers of sterile gauze, while solid digesta were sampled with tweezers. Fecal samples were collected aseptically from the rectum using disposable rectal exploration gloves. All samples were collected in duplicate, immediately snap-frozen in liquid nitrogen, and subsequently stored at −80°C. Samples of rumen content were collected for 6 days, while feces were sampled for an additional seventh day, to account for digestive passage rate.

#### DNA extraction, sequencing, and sequences analysis

A total of 108 samples were collected for both the PARL and digesta niches, and 126 samples were collected from feces. DNA was extracted using DNeasy PowerSoil Kit (Qiagen, Germany) with additional pre-processing steps for mechanical and enzymatic lysis (Neubauer et al., 2018). Details about the protocol are given in supplementary data in the Supplementary Material. The samples were sent to an external laboratory (Microsynth, Balgach, Switzerland) for targeted 16S rRNA gene sequencing. The primers 341F-ill (5′-CCTACGGGNGGCWGCAG-3′) and 802R-ill (5′-GACTACHVGGGTATCTAATCC-3′) were used to target the V3-V4 hypervariable regions of the bacterial 16S rRNA gene, with an expected product of approximately 460 bp (Klindworth et al., 2013). Libraries were prepared adding barcodes and Illumina adaptors through 16S Nextera two-step PCR. Equimolar pools of samples were sequenced using a 250 bp paired-end reads protocol for Illumina MiSeq sequencing platform. Demultiplexing, trimming of adaptors and reads merging was performed by Microsynth. Quality of the merged reads was inspected using FASTQC (Andrews and Babraham Bioinformatics, 2010), and the merged sequences were analyzed with software QIIME 2 (v. 2020.2) (Bolyen et al., 2019). Sequences were filtered for quality (PHRED score 20) before denoising with Deblur (Amir et al., 2017). Reads were trimmed at 385 nucleotides for rumen samples, which resulted in the loss of one sample from PARL (*n* = 107). Reads from fecal samples were trimmed at 400 nucleotides. All digesta and fecal samples passed the quality filtering and denoising (*n* = 108 and *n* = 126, respectively). The resulting tables were further filtered to exclude mitochondrial contamination. Taxonomy was assigned with a Naive Bayes classifier trained for the specific 16S rRNA gene target regions against the SILVA 132 99% OTU reference database (Quast et al., 2012). Chloroplasts were found with very low relative frequencies (<0.01%) in all three matrices analyzed. Alpha and beta diversity were calculated after samples with <1,000 reads were discarded (*n* = 105 for PARL, *n* = 107 for digesta and *n* = 121 for feces after filtering). The filtered amplicon sequence variants (ASVs) tables were used to calculate abundance-based coverage estimator (ACE) (Chao and Yang, 1993), Chao1 (Chao, 1984), Faith's phylogenetic diversity (Faith and Baker, 2006) and Shannon index (Shannon, 1948), as well as weighted and unweighted UniFrac distances (Lozupone et al., 2007) per each matrix. Phylogenetic Investigation of Communities by Reconstruction of Unobserved States 2 (PICRUSt2) was run using the QIIME2 plugin (v. 2019.10), with the default options (average NSTI was 0.18 for rumen samples and 0.36 for fecal samples) (Bolyen et al., 2019; Caicedo et al., 2020). Pathways names are reported according to MetaCyc Metabolic Pathway Database (Caspi et al., 2020).

### Statistical analyses

Datasets for ruminal pH, intake, microbial fermentation products and alpha diversity were checked for normal distribution with PROC UNIVARIATE procedure in SAS. When a variable was not normally distributed, PROC TRANSREG was run with Box-Cox model, to evaluate the best transformation to be applied. The presence of outliers was assessed calculating a simple linear regression with the PROC REG procedure, as well as Cook's distance (Cook's D) on the regression model and diagnostics on residuals. Values with a Cook's D above 0.8 were removed from the dataset for downstream analyses. Data were further analyzed using the PROC MIXED procedure of SAS with cow, experimental run, day, treatment and the interaction between day and treatment as fixed effects and cow within run as random effect. Cow within run was also considered as repeated measure, and *post-hoc* Tukey correction for *P*-values was applied. The animal as fixed effect was excluded from the model for the metabolomics data. Differences in beta diversity matrices (weighted and unweighted UniFrac distance) were calculated in QIIME2 using ADONIS (tested for adaptation day, treatment and their interaction). Analysis of the composition of microbiota was evaluated using Microbiome Multivariable Associations with Linear Models (MaAsLin2) package in R (Mallick et al., 2021). Differential abundance was calculated using Centered Log-Ratio (CLR) normalization and LM method, with adaptation day and treatment as fixed effects and individual animal and run as random effects. False Discovery Rate (FDR) was calculated with default parameters (Benjamini–Hochberg method) (Benjamini and Hochberg, 1995). Sequences were further analyzed applying the QIIME 2 plugin q2-longitudinal, performing a random forest regression aiming to predict the adaptation day on the basis of the microbiota composition (“longitudinal maturity-index”) (Bokulich et al., 2018). The group that did not receive the phytogenic feed additive was used as control, using 0.4 as fraction of samples to be excluded from the training set. PICRUSt2 results were analyzed using MaAsLin2, applying the same model used for the amplicon sequences. Metabolome data were analyzed using MetaboAnalyst (v. 5.0) to evaluate the effect of the progressive days and of the phytogenic feed additive on the rumen metabolite profile (Chong et al., 2019). Quantitative Enrichment Analysis was performed to identify enriched pathways during the days and due to the treatment. Spearman correlations were calculated between significantly affected taxa and metabolites as well as the 50 most abundant pathways detected *via* PICRUSt, using rcorr function of R package Hmisc (Harrell and Dupont, 2020). Further correlations were run between significantly affected genera and a subset of pathways selected within the dataset for their role in the metabolism of acetate, propionate, butyrate, ammonia, and lactate. Network analyses were performed through Model-based Integration of Metabolite Observations and Species Abundances 2 (MIMOSA2) web application (Noecker et al., 2016). Representative sequences for each ASV and absolute abundances of metabolites were used with PICRUSt KO genomes and KEGG metabolic model, with a similarity threshold of 0.99 and rank-based estimation. MIMOSA2 web application with such settings maps sequences to Greengenes database, which has not been updated since 2013. Previous research has demonstrated overall accordance between databases up to the family level (Sierra et al., 2020). Thus, results for network prediction are presented and discussed at the family level. Briefly, the MIMOSA2 framework estimates metabolic potential (CMP) scores based on the community composition, which variation is then compared to variation in measured metabolite abundances per each sample. Predictions of metabolite levels are validated through a regression model, and contributions are calculated as variation in metabolite abundance explained by a specific taxon. Contributions were considered significant with *P* ≤ 0.05 and FDR < 0.1. For all other statistical analyses, significance was considered at *P* ≤ 0.05 and tendencies were considered for 0.05 < *P* ≤ 0.10.

## Results

### Ruminal pH and feed intake

Results for feed intake and ruminal pH are given in detail in Table 1. Feed intake increased (*P* < 0.01) on the last days of experiment. Starch inclusion in the diet was steadily incremented, with starch intake gradually increasing from day 1 at 0.98 ± 0.12 kg to reach 3.55 ± 0.12 kg on day 6 (*P* < 0.01). Ruminal pH decreased with the progressive inclusion of concentrate in the diet (*P* < 0.01), with a minimum value below 5.8 on average on day 6 of experiment. Time with pH below 5.8 and acidosis index (determined by calculating the time that ruminal pH was below 5.8 per kg DMI) both increased along with the adaptation day (*P* = 0.01) (Khiaosa-ard et al., 2018).

**Table 1:** Nutrient intake and ruminal pH parameters during a 6-day adaptation to a high concentrate diet.

								*P*-values^3^		
**Intake, kg** ^**1**^	**Day 1**	**Day 2**	**Day 3**	**Day 4**	**Day 5**	**Day 6**	**SEM** ^**2**^	**Day**	**PHY**	Day*PHY
Dry matter	12.4	11.3	11.9	11.7^x^	13.3^y^	13.8	0.65	<0.01	0.97	0.73
Starch	0.98^a^	1.33^b^	1.85^a^	2.24^b^	3.05^a^	3.55^b^	0.12	<0.01	0.53	0.76
Non fiber carbohydrates	2.50	2.68^a^	3.24^b^	3.57^b^	4.53^a^^,^ ^x^	5.00^y^	0.20	<0.01	0.88	0.81
Neutral detergent fiber	6.16^a^	5.24^b^	5.11	4.60	4.79	4.50	0.28	<0.01	0.81	0.70
**Ruminal pH** ^**1**^										
Minimum pH	6.32^a^	6.10^b^	6.10^b^	5.98^a^	5.89^a^	5.76^b^	0.06	<0.01	0.71	0.95
Maximum pH	6.76	6.74^a^	6.58^b^	6.61^b^	6.48^a^	6.39	0.05	<0.01	0.56	0.71
Time below pH 5.8 (min/d)	33.3	2.50	18.3	128	161^a^	320^b^	68.9	0.01	0.23	0.49
Acidosis index^4^	2.35	0.21	1.14	2.07	3.39^a^	16.4^b^	5.41	0.01	0.61	0.82

Cows were divided in two groups supplemented or not with a phytogenic feed additive (PHY). The percentage of concentrate was incremented daily, starting from 10% on day 1 to reach 60% on day 6.

1 Data shown as least square means.

2 The largest standard error of the mean.

3 6P-values for the effect of day, phytogenic treatment (PHY) and the day × treatment interaction (Day^*^PHY).

4 Acidosis index was determined by calculating the time that ruminal pH was below 5.8 per kg DMI.

a, b Values with different superscripts indicate a significant difference (*P* ≤ 0.05) between consecutive days.

x, y Values with different superscripts indicate a tendency for difference (0.05 < *P* ≤ 0.10) between consecutive days.

### Microbiota alpha and beta diversity

#### Solid digesta

**Figure 1:**
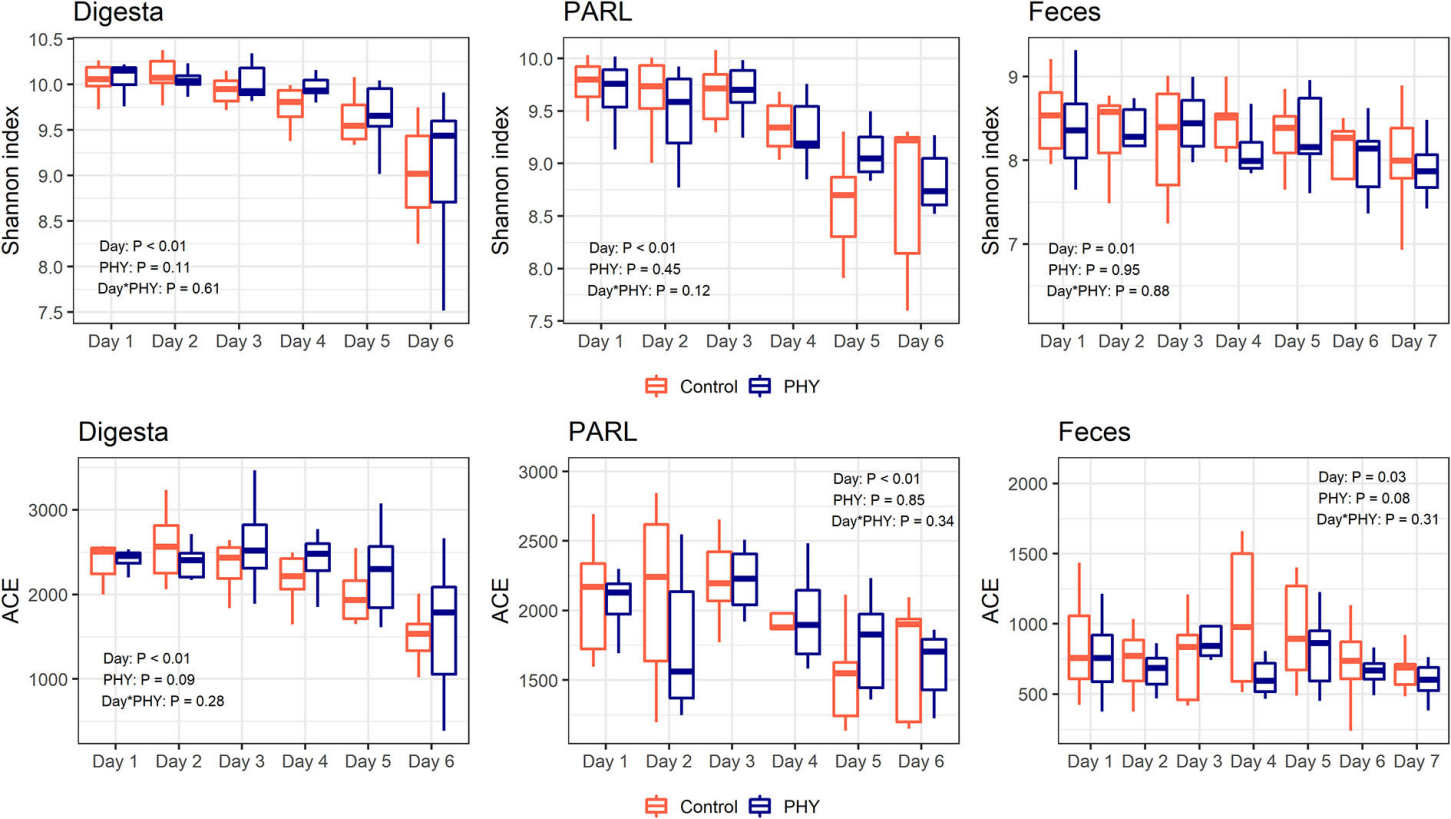
Alpha-diversity indices in the three analyzed matrices, for control and treatment (PHY) groups. Digesta, solid digesta; PARL, particle associated rumen liquid; ACE, Abundance-based Coverage Estimator. Samples were collected for six consecutive days for rumen content and for an additional seventh day for feces. *P*-values are presented for the effect of day, phytogenic treatment (PHY) and the day × treatment interaction (Day^*^PHY).

**Figure 2:**
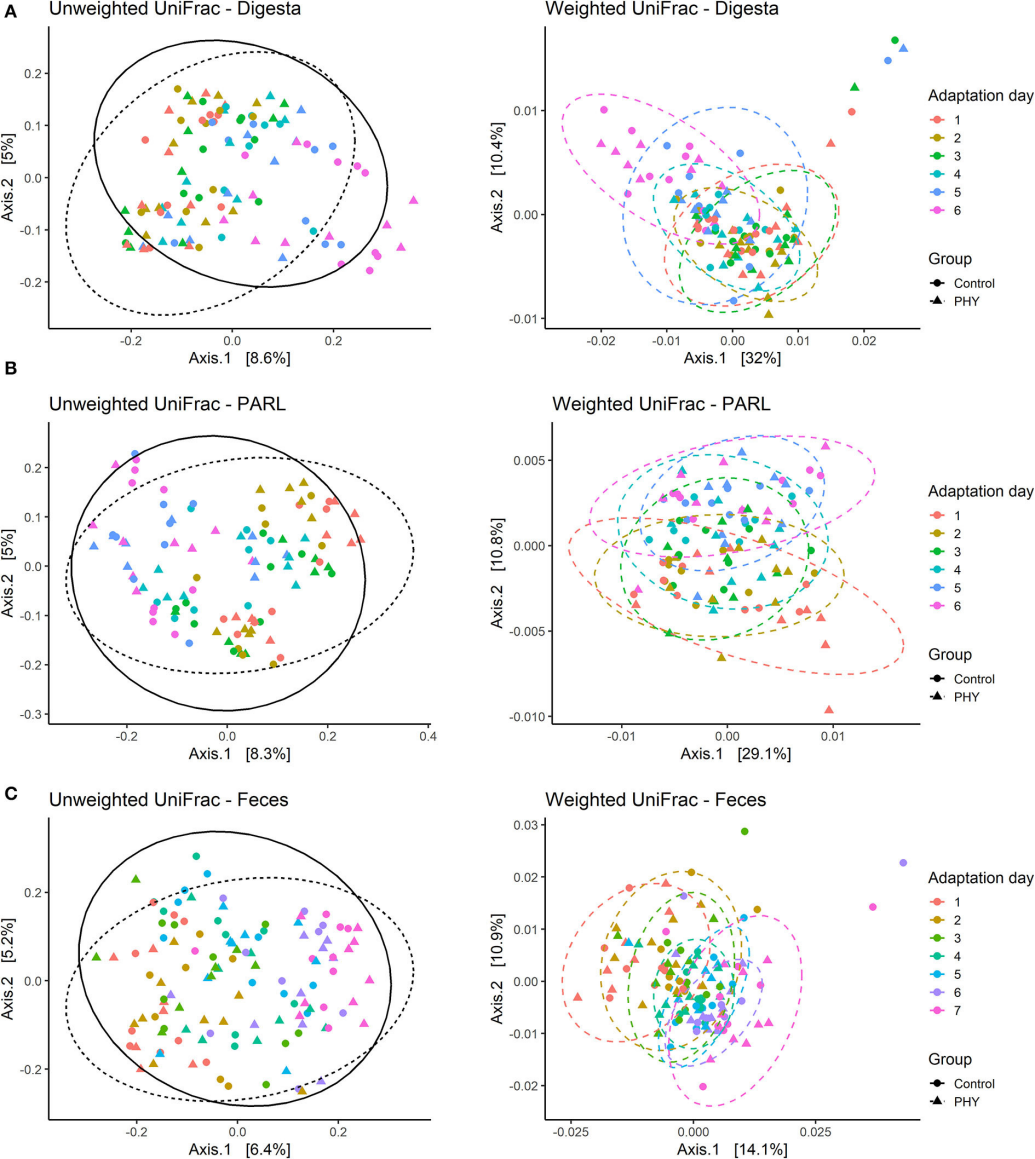
Beta-diversity for the three matrices analyzed. Graphs show weighted and unweighted UniFrac analysis over the experimental days for control and treatment (PHY) groups. **(A)** In solid digesta samples (Digesta), adaptation day impacted both weighted and unweighted UniFrac (*P* < 0.01), while the PHY treatment showed an effect only for the unweighted UniFrac (*P* < 0.01). **(B)** Beta-diversity in PARL was affected by the adaptation day (*P* < 0.01), while only unweighted UniFrac was affected by the PHY treatment (*P* = 0.03). **(C)** In feces, adaptation day had an effect on both beta diversity matrices (*P* < 0.01), while the treatment only showed a trend for unweighted UniFrac (*P* = 0.08).

ACE and Shannon index decreased to reach the minimum values on day 6 for both PHY and control group (Figure 1). All other alpha diversity parameters evaluated were affected by the adaptation day (*P* < 0.01) (Supplementary Table S2). The PHY showed an effect for ACE, Chao1, and Faith's phylogenetic diversity (*P* = 0.09, *P* = 0.09 and *P* = 0.03, respectively). ADONIS revealed a significant impact of the adaptation day on both weighted and unweighted UniFrac analyses, while the PHY treatment showed an effect only for the unweighted UniFrac (*P* < 0.01) (Figure 2A). There was no significant effect for the interaction between the PHY and the adaptation days.

#### PARL

Alpha diversity indexes in PARL decreased over the adaptation days (*P* < 0.01), with lower values reached on day 5 (Figure 1; Supplementary Table S2). The PHY treatment did not affect the alpha diversity. Beta diversity in PARL was affected by the adaptation day (*P* < 0.01), while Unweighted UniFrac was affected by the PHY treatment (*P* = 0.03) (Figure 2B). The interaction between PHY and adaptation day was not significant.

#### Feces

Alpha diversity was affected by the adaptation day (Figure 1), with values decreasing over the 7 days for both groups (Supplementary Table S3). The PHY affected both ACE and Chao1 indexes (*P* = 0.08). ADONIS for beta diversity in feces revealed an effect of the adaptation day for both distance matrices (*P* < 0.01), but no effect of the interaction between PHY and day. The PHY treatment affected only unweighted UniFrac, showing a tendency (*P* = 0.08) (Figure 2C).

### Microbiota composition and differential abundance

#### Solid digesta

**Figure 3:**
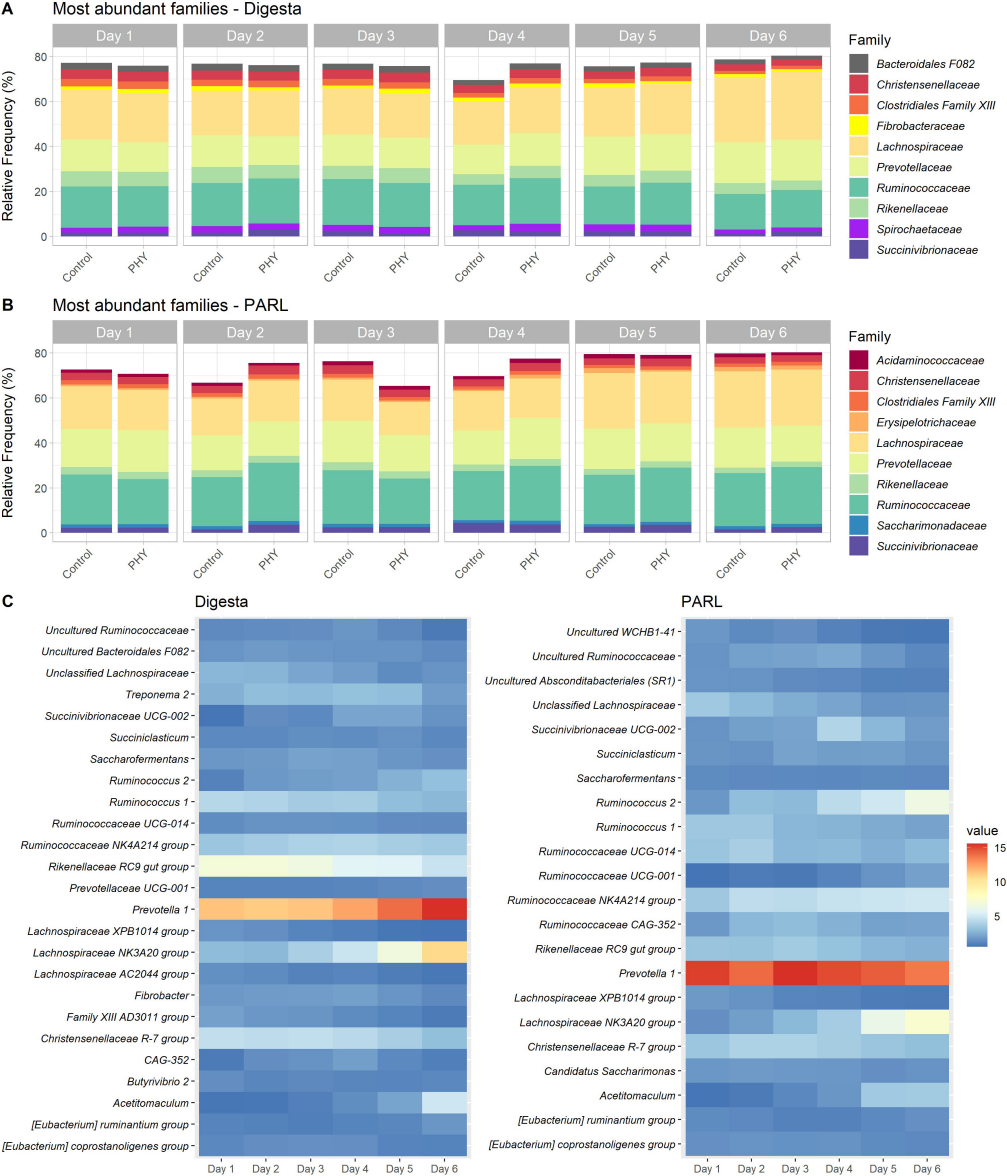
Microbiota composition of the rumen samples. Bar plots showing the mean relative frequency of the 10 most abundant families (across all samples) in **(A)** solid digesta and **(B)** particle associated rumen liquid (PARL) over the experimental days and between the two experimental groups (PHY and control). The heatmaps **(C)** show the mean relative frequency of the 25 most abundant genera across all samples for solid digesta (Digesta) and particle associated rumen liquid (PARL) over the experimental days.

A total of 4,148,399 reads were grouped into 19,324 features and assigned to 21 phyla, of which the two most abundant were *Firmicutes* and *Bacteroidetes*, which accounted together for more than 80% of all the reads (Supplementary Figure S1). The most represented families are presented in Figure 3A. *Prevotella* 1 (12.7%) was the most abundant genus on average across all samples, followed by *Rikenellaceae* RC9 gut group (6.1%) and *Lachnospiraceae* NK3A20 group (5.4%) (Figure 3C). Most phyla were significantly affected by the increasing amount of concentrate in the diet starting from day 5, with the highest frequency of *Firmicutes* (59.9%) on day 6 (*P* < 0.01) and of *Bacteroidetes* on day 5 (30.5%) (*P* < 0.01). Effects at the family level were appreciable mainly from day 4. Family *Lactobacillaceae* decreased over the 6 days (*P* = 0.03), while *Erysipelotrichaceae* tended to increase from day 4 (*P* < 0.01). *Lachnospiraceae, Prevotellaceae* and *Ruminococcaceae* were also affected by the concentrate included in the diet, with the first two families increasing by day 6 (*P* < 0.01) and the latter decreasing (*P* < 0.01). Genera *Prevotella* 1 (*P* = 0.08), *Lachnospiraceae* NK3A20 group (*P* < 0.01), *Ruminococcus* 2 (*P* = 0.02), and *Selenomonas* 1 (*P* < 0.01) increased over the experimental days. *Ruminococcaceae* NK4A214 group increased over the first 4 days (4.1%; *P* < 0.01) and decreased again on day 6 (3.6%; *P* < 0.01). Similarly, *Lachnospiraceae* XPB1014 group, ND3007 group, and NK4A136 group decreased over the experimental days, from day 5 (*P* < 0.01, *P* < 0.01, and *P* = 0.01, respectively). The PHY treatment increased the relative frequency of *Ruminococcaceae* UCG-005 (0.28 and 0.20% in PHY and control group, respectively; *P* = 0.01) and *Ruminococcaceae* V9D2013 group (0.07 and 0.05% in PHY and control group, respectively; *P* = 0.04). *Ruminiclostridium* 9 (0.12 and 0.21% in PHY and control group, respectively; *P* = 0.10) and *Alloprevotella* (0.02% and 0.03% in PHY and control group, respectively; *P* = 0.08) tended to be more abundant in the control group. The 50 most important features detected with the maturity index prediction were used to build a heatmap showing the trend for their frequency over the 6 days of sampling (Supplementary Figure S3A). Among these, more than half were assigned to families *Lachnospiraceae* and *Ruminococcaceae*, and in particular to genera *Lachnospiraceae* NK3A20 group and *Ruminococcaceae* NK4A214 group (Supplementary Table S7). Both genera were also identified as differentially abundant, together with *Prevotella* 1 and other *Lachnospiraceae* genera (XPB1014 group, ND3007 group, NK4A136 group).

#### PARL

For PARL, 4,493,095 reads were grouped into 18,557 features. A total of 24 phyla were detected, and as for digesta, the two most abundant were *Firmicutes* and *Bacteroidetes* (Supplementary Figure S1). Figure 3B shows the relative abundance of the most abundant families. *Prevotella* 1 (16.1%) and *Lachnospiraceae* NK3A20 group (4.2%) were among the most abundant genera on average across all samples, together with *Ruminococcaceae* NK4A214 group (4.8%) (Figure 3C). The progressive inclusion of concentrates in the diet significantly affected the relative abundance of phyla and of families, mainly from day 3. Family *Lactobacillaceae* and genus *Lactobacillus* decreased over the days (*P* < 0.01), while *Streptococcaceae* increased only on day 6 (0.11%, *P* = 0.05). Similarly, *Succinivibrionaceae* tended to increase only on day 5 (*P* = 0.06). Families *Lachnospiraceae, Ruminococcaceae*, and *Prevotellaceae* showed fluctuations over the experimental days. Genus *Prevotella* 1 started to decline on day 4 (16.4%) to day 5 (15.9%) (*P* = 0.03), while *Prevotellaceae* UCG-001 and *Prevotellaceae* NK3B31 group increased over the experimental days (*P* < 0.01). *Lachnospiraceae* NK3A20 group and *Ruminococcaceae* NK4A214 group's relative frequency increased from day 2 (*P* = 0.01 and *P* < 0.01, respectively). *Burytivibrio* 2 and *Pseudobutyrivibrio* increased from day 5 (*P* = 0.03 and *P* < 0.01, respectively). The PHY supplementation had more impact on PARL than digesta microbiota composition: 14 families and 36 genera were significantly affected by the treatment. Families *Veillonellaceae* (*P* < 0.01) and *Clostridiales* Family XI (*P* < 0.01) had higher relative frequency in the control group. The PHY treatment increased the frequency of genera *Ruminococcaceae* UCG-011 (1.1 and 0.8% in PHY and control group, respectively; *P* < 0.01), *Ruminococcaceae* UCG-005 (0.3% and 0.2% in PHY and control group, respectively; *P* = 0.02), and *Lachnospiraceae* UCG-006 (0.13% and 0.11% in PHY and control group, respectively; *P* = 0.04). Supplementary Figure S3B shows the 50 most important taxa, identified with the random forest regression and their variation over the sampling days per each group, control or treatment. As for digesta, most of the features were classified as belonging to families *Lachnospiraceae* and *Ruminococcaceae* (Supplementary Table S7). Features classified as *Prevotella* 1, *Butyrivibrio* 2, and *Lactobacillus* were identified as part of the key taxa in the adaptation to the new diet both by the random forest and the differential expression analysis.

#### Feces

**Figure 4:**
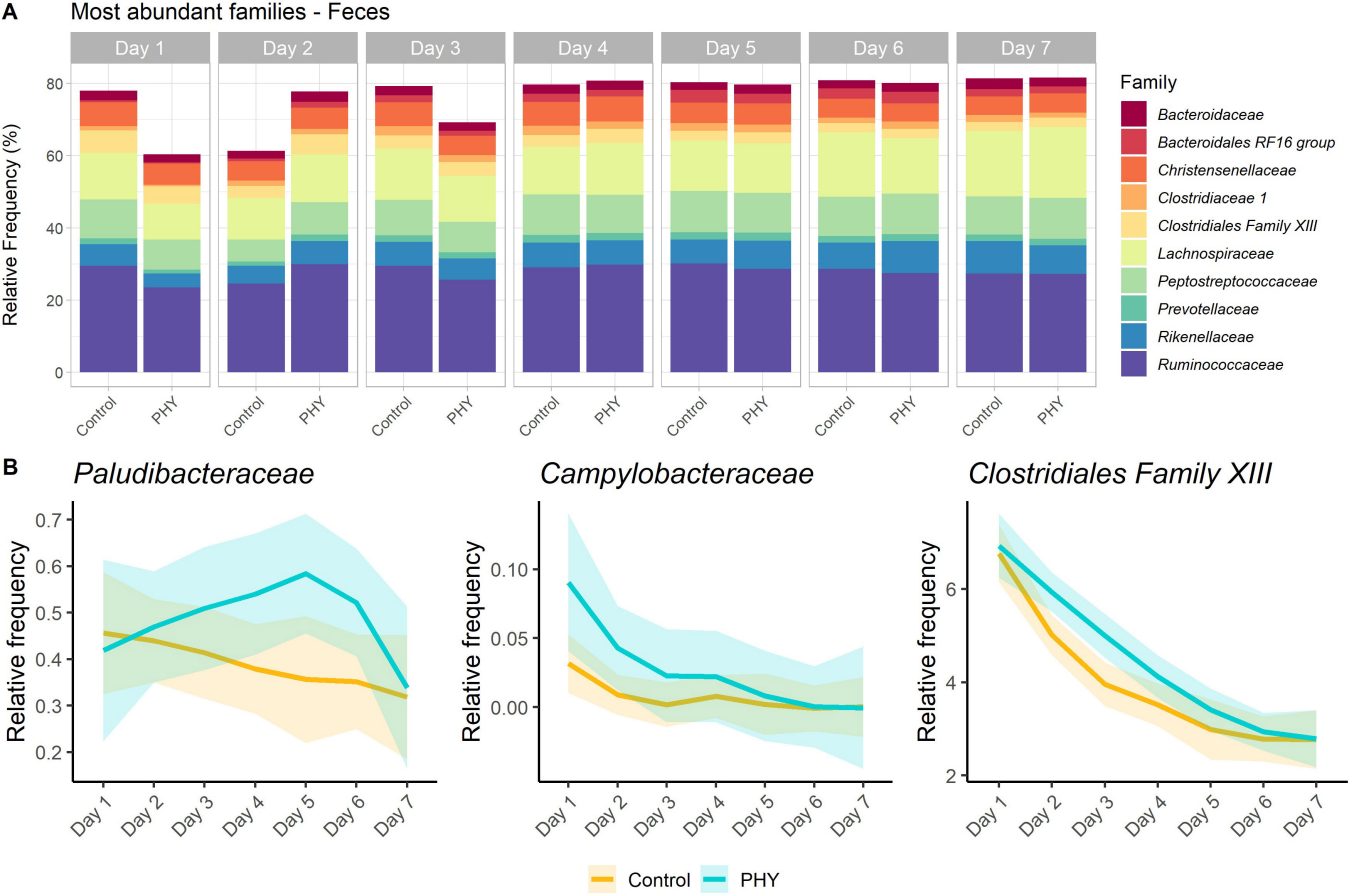
Microbiota composition of the fecal samples. **(A)** Barplot showing the mean relative frequency of the 10 most abundant families (across all samples) over the experimental days and between the two experimental groups (PHY and control). **(B)** Plots showing the different relative frequency trends over the experimental days between the treatment (PHY) and control group. The PHY increased the relative abundance of *Paludibacteriaceae* (*P* = 0.06), *Campylobacteraceae* (*P* = 0.09) and *Clostridiales Family XIII* (*P* = 0.09).

Reads for fecal samples (1,426,602) were assigned to 10,211 features. Features were grouped in 15 phyla, of which only three had a relative abundance above 1% (*Firmicutes, Bacteroidetes* and *Spirochaetes*, 76.9, 17.7, and 1.3%, respectively) (Supplementary Figure S1). Most represented families are shown in Figure 4A. *Ruminococcaceae* UCG-005, *Romboutsia* and *Christensenellaceae* R-7 group were the most abundant genera on average across all samples (8.4, 7.2, and 6.7%, respectively) (Supplementary Figure S2). Effects on phyla were mostly visible from day 3 or 4, while *Tenericutes* showed an increase only on day 7 (*P* < 0.01). Phylum *Epsilonbacteraeota*, to which family *Campylobacteraceae* belongs, tended to decrease on day 5 (*P* = 0.06), disappearing in the last 2 days. Most of the families were significantly affected by the diet changes from day 3. Family *Ruminococcaceae* tended to decrease over the 7 days, significantly from day 5 (*P* < 0.01). In parallel, *Lachnospiraceae* increased from 14.3% on day 1 to 21% on day 7 (significant increment from day 3, *P* = 0.05). The progressive inclusion of concentrate in the diet affected 109 genera. Genera *Lachnospiraceae* AC2044 group (*P* = 0.02), *Ruminobacter* (*P* = 0.04), *Blautia* (*P* = 0.03), *Butyrivibrio* (*P* = 0.01), *Treponema* 2 (*P* < 0.01), and *Ruminococcus* 2 (*P* = 0.03) increased already from day 2. *Ruminococcus* 1 decreased on day 2 (0.4%; *P* = 0.02) and increased again on day 6 (1.4%; *P* = 0.04). The PHY supplementation tended to increase the abundance of families *Paludibacteriaceae, Campylobacteraceae*, and *Clostridiales* Family XIII in the fecal samples (Figure 4B). At the genus level, the PHY treatment increased the abundance of low abundant taxa, such as *[Eubacterium] ventriosum* group (0.04% and 0.02% in PHY and control group, respectively; *P* < 0.01), *Prevotellaceae* UCG-003 (0.6% and 0.3% in PHY and control group, respectively; *P* < 0.01), *Lachnospiraceae* UCG-007 (0.08% and 0.02% in PHY and control group, respectively; *P* = 0.02), and *Ruminococcaceae* UCG-011 (0.08% and 0.04% in PHY and control group, respectively; *P* = 0.06). Random forest regression (Supplementary Figure S3C) identified the key component of the fecal microbiota as mainly belonging to families *Lachnospiraceae* and *Ruminoccaceae* (Supplementary Table S7). Family *Clostridiales* Family XIII and genus *Prevotellaceae* UCG-003 were confirmed to play an important role in the adaptation to a new diet by both the random forest and the differential abundance analysis.

### Microbial activity parameters

The concentration of total VFAs was impacted by dietary adaptation day (Table 2, *P* < 0.01). Acetate, propionate, and butyrate concentration increased over the 6 days of experiment (*P* < 0.01), reaching the highest values on day 5. Isobutyrate and isovalerate concentrations decreased from day 1 to day 6 (*P* < 0.01). There was an interaction between PHY treatment and experimental day for propionate concentration (*P* = 0.04), and a tendency toward interaction for total VFA concentration (*P* = 0.08). Ammonia concentration in the rumen fluctuated over the days (*P* = 0.05). Values tended to increase on day 2, to decrease again on day 3, reaching the lowest in the PHY group (19.22 ± 3.12 mg/dl). Ammonia concentration tended to be higher on day 6 for the PHY group, with a tendency for an interaction between days and treatment (*P* = 0.06). D-Lactate concentration showed high numerical concentrations on days 4 and 5, but no significant effects of diet or treatment.

**Table 2:** Concentration of volatile fatty acids (VFA), ammonia, and D-lactate measured in the rumen fluid.

	**Day 1**		**Day 2**		**Day 3**		**Day 4**		**Day 5**		**Day 6**			***P*** **-values** ^**3**^		
**LSM^1^**	**CON**	**PHY**	**CON**	**PHY**	**CON**	**PHY**	**CON**	**PHY**	**CON**	**PHY**	**CON**	**PHY**	**SEM^**2**^**	**Day**	**PHY**	Day*PHY
Total VFA, mM	105^a^	84.2^a^	121^b^	127^b^	143	124	147^x^	131^x^	163^y^^,^ ^a^	153^y^^,^ ^a^	128^b^	142^b^	7.26	<0.01	0.11	0.08
Acetate, mM	67.9^a^	59.1^a^	77.9^b^	81.8^b^	92.2	81.6	94.7	86.5	96.0^a^	96.0^a^	75.0^b^	84.5^b^	5.19	<0.01	0.42	0.17
Propionate, mM	18.2^a^	14.3^a^	20.8^b^	22.4^b^	27.0	22.6	27.3^x^	22.8^x^	30.7^y^	27.1^y^	25.2	27.4	1.56	<0.01	0.12	0.04
Butyrate, mM	11.8^a^	9.21^a^	13.9^b^	14.7^b^	15.5	13.0	17.3^a^	14.9^a^	24.0^b^	21.9^b^	20.4	23.6	1.09	<0.01	0.17	0.11
Valerate, mM	2.14^a^	1.89^a^	2.34^b^	2.48^b^	2.77	2.31	2.56	2.44	2.55	2.73	2.39	2.57	0.18	0.01	0.71	0.12
Isovalerate, mM	2.58^x^	2.46^x^	2.84^y^	2.93^y^	2.69	2.40	2.40	2.35	2.52^a^	2.62^a^	1.90^b^	2.04^b^	0.21	<0.01	0.90	0.68
Isobutyrate, mM	1.89^x^	1.72^x^	1.98^y^	2.12^y^	1.88	1.77	1.64	1.71	1.75^a^	1.77^a^	1.37^b^	1.33^b^	0.14	<0.01	0.92	0.40
Ratio acetate:propionate	3.71	3.86	3.74	3.70	3.43	3.69	3.45^a^	3.84^a^	3.02^b^	3.55^b^	3.01	3.18	0.16	<0.01	0.10	0.40
Ammonia, mg/dL	26.5	25.8	28.6	32.7	27.6	19.2	29.7	24.1	26.8	27.2	16.5	26.5	3.12	0.05	0.99	0.06
D-Lactate, mM	0.11	0.12	0.07	0.07	0.08	0.11	0.11	0.24	0.20	0.09	0.03	0.15	0.16	0.27	0.20	0.14

Values were measured 4 h after the morning feeding, during adaptation to a high concentrate diet. The animals were divided in two groups, of which one received a phytogenic feed additive (PHY), while the other served as control (CON).

1 Least square means.

2 The largest standard error of the mean.

3 P-values for the effect of day, phytogenic treatment (PHY) and the day × treatment interaction (Day*PHY).

a, b Values with different superscripts indicate a significant difference (*P* ≤ 0.05) between consecutive days.

x, y Values with different superscripts indicate a tendency for difference (0.05 < *P* ≤ 0.10) between consecutive days.

**Figure 5:**
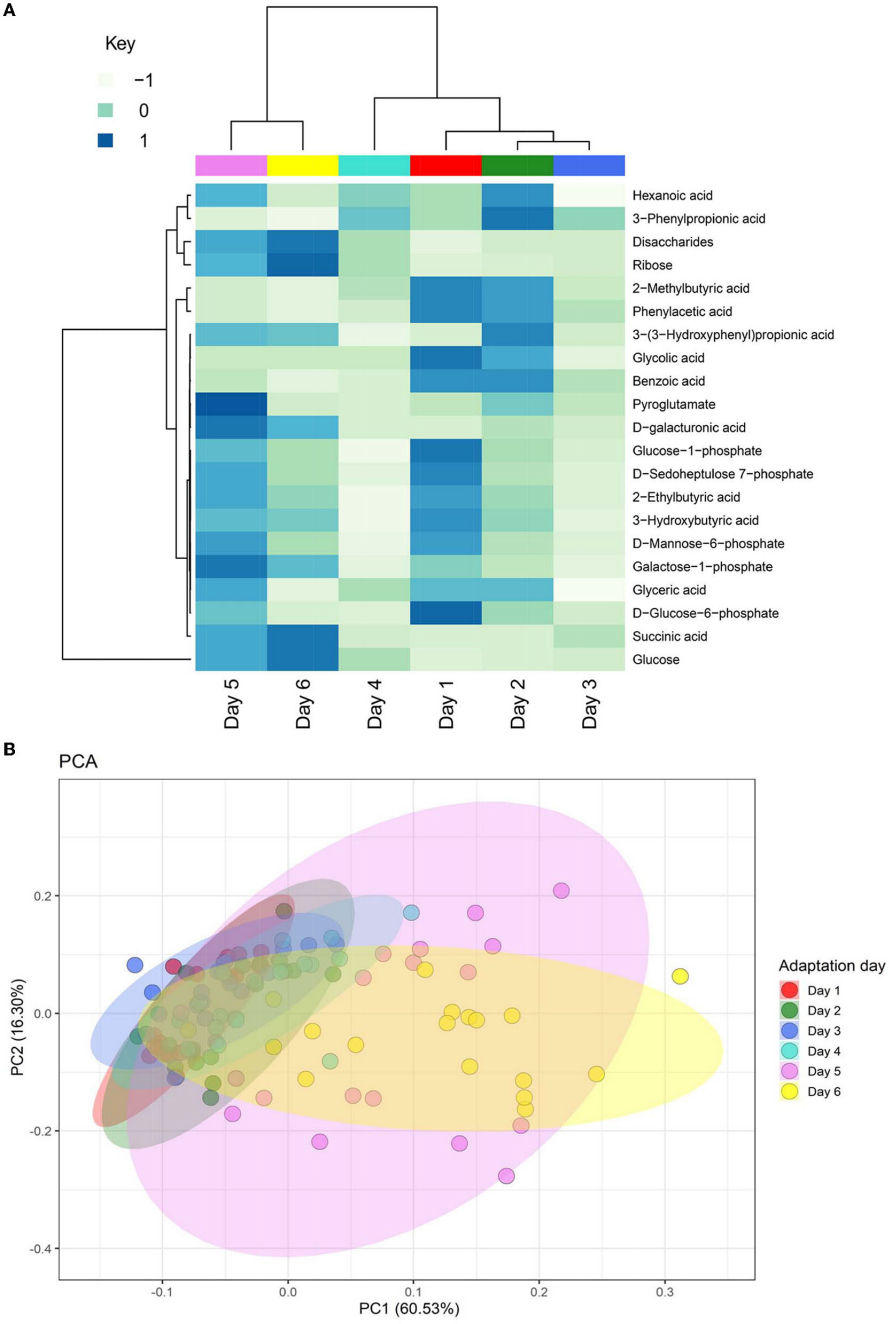
Heatmap **(A)** showing the change in concentration of metabolites measured in the rumen fluid across the six experimental days. Rows were scaled to have mean zero and standard deviation one, to normalize the different concentrations of each metabolite. Values range from −1 (lowest concentration measured) to 1 (highest concentration measured). The colors below the dendrogram correspond to the experimental days. The dendrogram shows a separate cluster for the last two days of experiment (days 5 and 6). Principal Component Analysis (PCA) **(B)** showing the variance of the metabolite composition in the rumen fluid. The graph is based on the normalized values calculated in MetaboAnalyst over the six experimental days.

**Figure 6:**
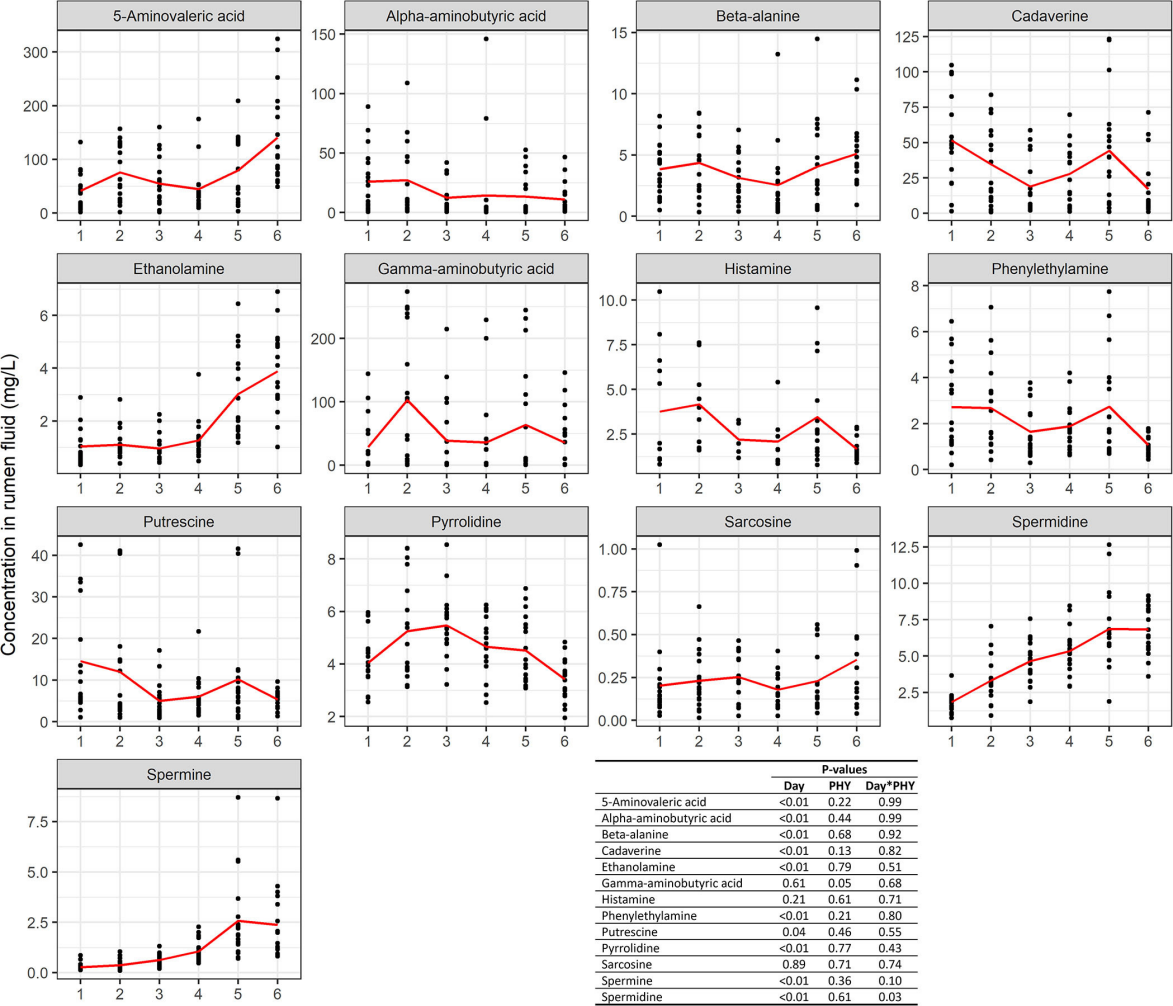
Concentration of biogenic amines measured in the rumen fluid 4 h after the morning feeding. The red line represents the trend of the mean values over the 6 days of experiment. *P*-values are presented for Day, treatment (PHY) and interaction between Day and treatment (Day*PHY).

All the metabolites measured were affected by the dietary adaptation day (Figure 5A). There was an interaction between PHY treatment and adaptation day for 3-hydroxybutyric acid (*P* = 0.01) and disaccharides (*P* = 0.01). Furthermore, there was a trend for interaction for ribose and galactose-1-phosphate (*P* = 0.08). 3-(3-hydroxyphenyl) propionic acid tended to be affected by the treatment (*P* = 0.09) (Supplementary Table S4). Principal component analysis (PCA) of the metabolites showed a clear separation of the last 2 days of adaptation from the previous four, as shown in Figure 5B. This variance was explained mainly by three compounds: acetic acid, butyric acid, and glucose. The same compounds, with the addition of propionic acid, were found to be important features for the discrimination between PHY and control group, although the groups did not cluster separately. A total of 13 biogenic amines were identified and analyzed in the rumen fluid. The majority were affected by the adaptation day, with the exception of histamine, sarcosine, and gamma-aminobutyric acid (Figure 6). The latter showed considerably lower concentrations in the PHY group compared to the control (*P* = 0.05). There was an interaction between adaptation day and PHY treatment for spermidine (*P* = 0.03).

### Pathways prediction analysis

Quantitative enrichment analysis showed effects of the day on several pathways, notably phenylalanine metabolism, pentose phosphate pathway, glycolysis/gluconeogenesis, starch and sucrose metabolism, and galactose, fructose and mannose metabolism (*P* < 0.01). The PHY treatment showed some tendencies, specifically for propanoate metabolism, glycolysis/gluconeogenesis, glyoxylate and dicarboxylate metabolism and pyruvate metabolism (*P* = 0.10).

**Figure 7:**
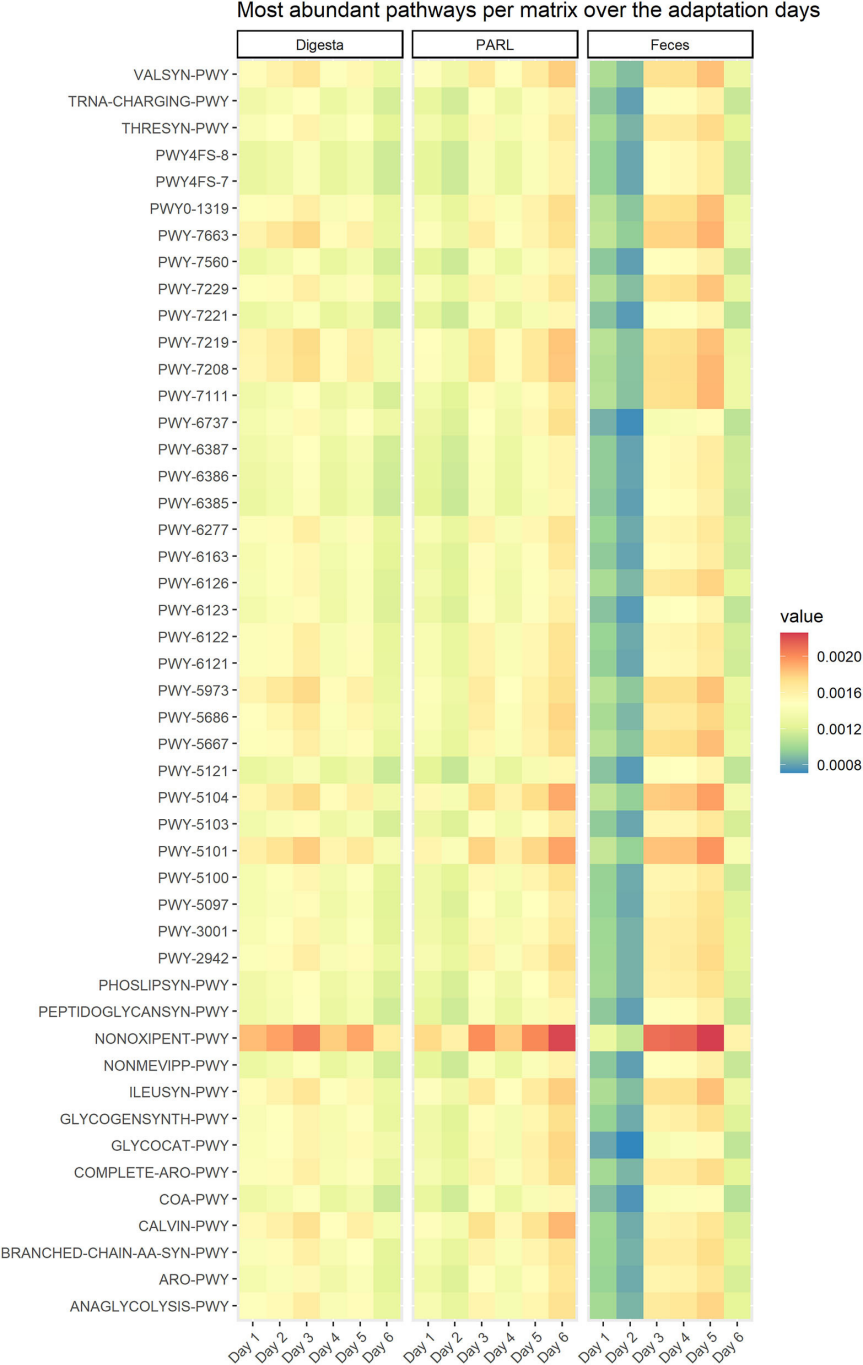
Relative abundance of the most abundant pathways for the three analyzed matrices. Pathways were predicted *via* PICRUSt2 based on the 16S rRNA gene amplicon sequencing of samples of solid digesta (Digesta), particle associated rumen liquid (PARL) and feces.

The relative abundances of the most abundant pathways predicted by PICRUSt which were shared by all three matrices for the 6 days of experiment are shown in Figure 7. All most abundant pathways were affected by the concentrate inclusion in the diet, starting from day 2 in PARL and from day 4 or 5 in digesta. Similarly, in feces, changes in the 50 most abundant pathways were evident from day 3 or 4. The PHY treatment affected 13, 46, and 12 pathways in digesta, PARL, and feces, respectively. Pathways PWY-7013 [(S)-propane-1,2-diol degradation], PWY-5676 (acetyl-CoA fermentation to butanoate II), and PWY-6588 (pyruvate fermentation to acetone) were enhanced by the treatment both in digesta and PARL samples. Pathway P163-PWY (L-lysine fermentation to acetate and butanoate) was affected by the PHY additive in all three matrices investigated, being more expressed in the PHY group in digesta and PARL, but less expressed in feces.

### Network analysis

**Figure 8:**
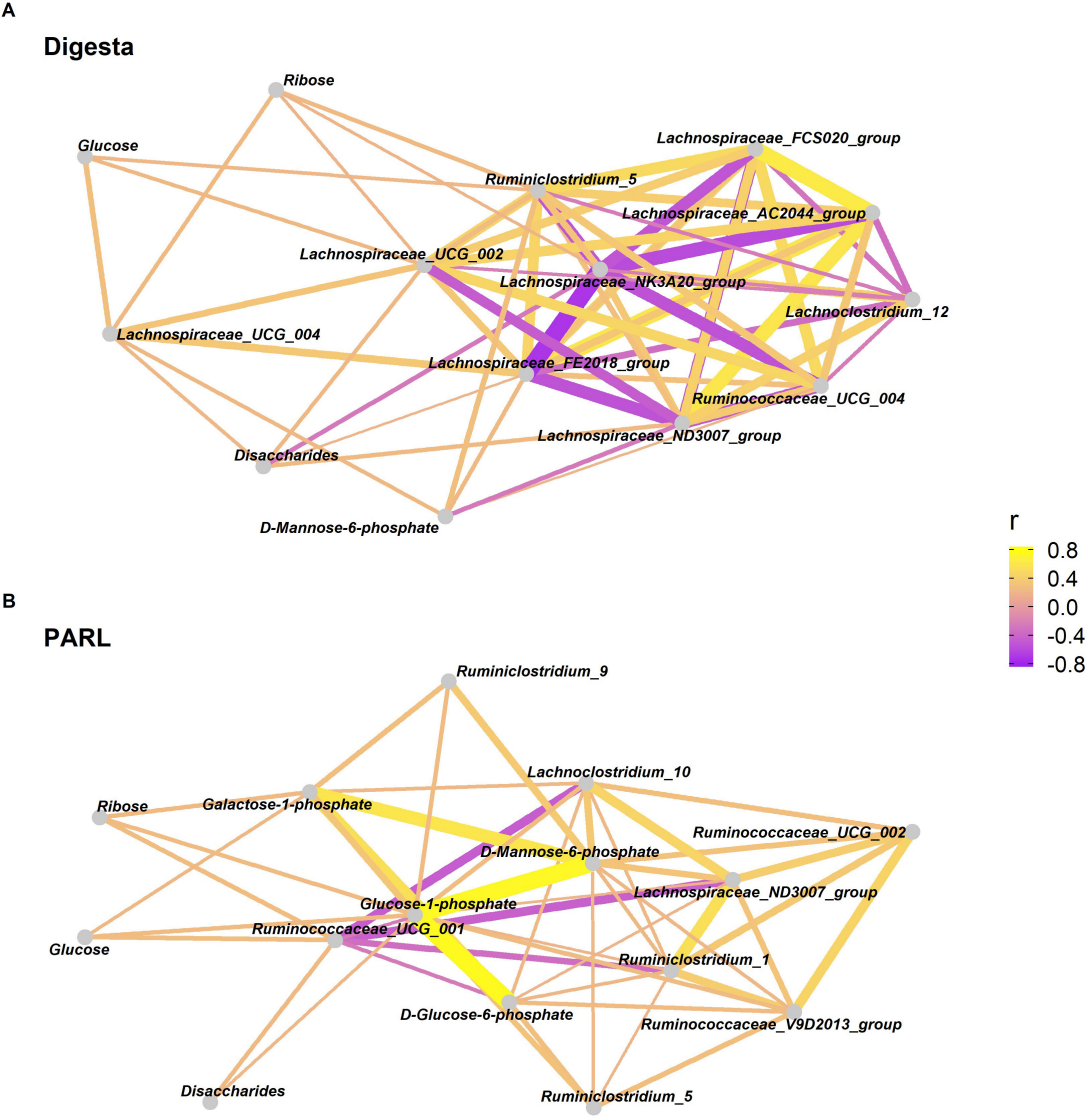
Network analysis based on Spearman correlations between carbohydrates and microbes in the rumen. Correlations were calculated between genera belonging to families *Lachnospiraceae* and *Ruminococcaceae* and carbohydrates in **(A)** solid digesta (Digesta) and **(B)** particle associated rumen liquid (PARL). Correlation coefficients are indicated by color (purple = negative, yellow = positive) and line thickness.

Spearman correlation between differentially abundant taxa and metabolites revealed positive correlations between members of the family *Lachnospiraceae* and *Ruminococcaceae* and carbohydrates, both in digesta and in PARL samples, although with relatively low R scores (Figure 8). Three genera identified in digesta samples (*Streptococcus, FD2005* and *Lachnobacterium*) were positively correlated with D-lactate concentration measured in the rumen fluid, although with low R scores (*P* < 0.01; R scores = 0.55, 0.30, and 0.37, respectively). The same genera were also correlated with the pathways associated with lactate, such as ANAEROFRUCAT-PWY [homolactic fermentation (fructose fermentation to lactate)], P122-PWY [heterolactic fermentation (lactate heterofermentation)], P124-PWY [*Bifidobacterium* shunt (glucose fermentation to lactate (*Bifidobacteria*)], P461-PWY (hexitol fermentation to lactate, formate, ethanol and acetate), PWY-5100 (pyruvate fermentation to acetate and lactate II), and PWY-6641 (superpathway of sulfolactate degradation) (Supplementary Figure S4).

For digesta, MIMOSA2 calculated the CMP scores for five metabolites, including acetate, D-Mannose 6-phosphate, and phenylacetic acid, for a total of 1243 taxa involved (Supplementary Table S5). In PARL samples, a total of 1061 taxa were found to be contributing to the variation of four metabolites, including succinate and sedoheptulose 7-phosphate (Supplementary Table S6). CMP scores were calculated also for D-glucose 6-phosphate and lactate in both PARL and digesta. Both ruminal niches hosted *Ruminococcaceae* and *Succinivibrionaceae* as major taxa driving differences in lactate concentration across samples, both encoding for K01069 (hydroxyacylglutathione hydrolase), which contributes to form D-lactate from carbohydrate metabolism. However, no taxon could explain more than 1% of the variance for lactate. The variation in D-glucose 6-phosphate concentration appeared to be driven mostly by *Erysipelotrichaceae* and *Ruminococcaceae* in PARL samples. In digesta samples, most of the variance for this metabolite were explained by *Lachnospiraceae*, followed by *Erysipelotrichaceae* and *Ruminococcaceae*. The variation in D-mannose-6-phosphate concentration was mainly contributed by *Erysipelotrichaceae, Ruminococcaceae, Lachnospiraceae*, and *Coriobacteriaceae*. The taxa most associated with variation in glucose-1-phosphate concentration were *Ruminococcaceae* and *Lachnospiraceae*, despite explaining <1% of the variation. In PARL, families *Prevotellaceae* and *Succinivibrionaceae* were identified as the main taxa putatively responsible for the variation of sedoheptulose-7-phosphate, through K03271 (D-sedoheptulose-7-phosphate isomerase). Three biogenic amines (spermidine, 5-aminovaleric acid, and spermine) were associated with 1,226 taxa in digesta samples, and with 1,155 taxa in PARL. In addition, significant associations were found in PARL samples also for beta-alanine, putrescine, cadaverine, and phenethylamine (Supplementary Table S6). In both niches, *Ruminococcaceae* and *Lachnospiraceae* were putatively responsible for the main variation for all the biogenic amines, except for putrescine and phenethylamine, for which the main producers were identified as *Methanobacteriaceae* and *Anaerolinaceae*, respectively.

## Discussion

### Rapid adaptive response of the microbiota

The aim of this study was to evaluate the day-by-day adaptation of the gastrointestinal microbiota in cattle fed increasing amounts of readily fermentable carbohydrates, and the effects of diet supplementation with a blended PFA, through the analysis of composition, metabolism, and predicted pathways. These results will help to elucidate the mechanisms of adaptation of microbiota undertaken when challenged with a dietary change, particularly if there is a critical point in the adaptive response, over where the system becomes unbalanced and changes toward dysbiosis (Sommer et al., 2017).

The gradual inclusion of starch in the diet and the phytogenic additive altered microbiota composition and metabolic activity in all GIT niches evaluated in our experiment. Previous studies have not shown day-to-day alterations of the microbial composition in rumen content nor in feces, whereas we demonstrated a rapid adaptation of the microbiota to changing feeding conditions (Plaizier et al., 2017; Huang et al., 2020). This could be due to the different feeding programs of the animals, or the sampling schedule employed in the other experiments. While other studies looking at the difference between days have allowed between 7 and 40 days of adaptation prior to sampling, in our experiment the goal was to assess the impact of dietary change and therefore, diet did not remain constant throughout the experiment. This unique look at the process of microbial adaptation allow for new insights into the evolution of SARA and potential points for modulating ecological shifts to improve animal health. However, we monitored the microbiota over the first 6 days of adaptation to a new diet, and it is recognized that further adaptations of the microbiota are likely after this time, as the community fully adapts to the diet over the following days.

### Niche-specific responses

Interestingly, PARL was the quickest and most responsive to dietary changes, with the highest number of taxa and predicted pathways affected by the increasing concentrate intake and the phytogenic additive. Despite the overall similarities in microbial composition, our results suggest a different metabolic activity occurring in PARL and in solid digesta. Variation in reaction to dietary challenges in the different ruminal niches is a well-known phenomenon (Metzler-Zebeli et al., 2015; Schären et al., 2017). It is suggested that the differences in bacterial activity in the rumen, within the same phylum and family, could be due to the accessibility of the substrates, depending on the solubility of the ingested material (Rubino et al., 2017; Hart et al., 2018). Thus, the macroscopic differences between solid and liquid digesta could explain the different responses to the high concentrate feeding found in our study. Differences detected in the time of reaction to the newly introduced substrates between the two niches suggest a slower adaptation of solid digesta to dietary changes, probably due to the microcolonies formed by the bacteria on the fiber particles in the ruminal mat, causing them to be more refractory to rapid adjustments (Cheng et al., 1981).

It is recognized that the microorganisms that colonize the distinct sections of the GIT are substantially different, but we found some similarities in the adaptation to the dietary challenge between the ruminal and the fecal microbiota (Ozbayram et al., 2018; Holman and Gzyl, 2019), with a delay in the changes for the hindgut. As expected, the increase in concentrate intake reduced the alpha diversity indices for all three matrices analyzed. Reduced richness and diversity are a common finding in cattle-fed high-concentrate diet and it is believed that such a reduction could limit the microorganisms' capacity of resources utilization (Plaizier et al., 2017). However, it has previously been suggested that a reduction in diversity can actually increase the efficiency in utilization of substrates in ruminants (Shabat et al., 2016; Belanche et al., 2019), and our findings confirm that the gastrointestinal microbiota can rapidly adapt its metabolic capability to face newly available substrate, despite the species diversity reduction.

Since the total mean retention time of ingested feed in cattle is at least 24 h, it was expected that changes in microbial activity and composition in feces would be evident with ~1 day of delay in respect to the ruminal environment (Hartnell and Satter, 1979; Mambrini and Peyraud, 1997). This parallel shaping of the microbial population in the rumen and in feces is well shown by the beta diversity graphs, in which separated clusters are noticeable for the last 2 days of experiment, being days 5 and 6 for the ruminal niches and days 6 and 7 for the fecal samples.

While Plaizier et al. (2017) found the composition of the fecal microbiota to be relatively stable over time during SARA, we demonstrated a daily adaptation of fecal microorganisms to the increment in readily digestible concentrate in the diet, with altered composition and activity. Nevertheless, some researchers suggest that the response of the large intestine to high-concentrate feeding might be not consistent among experiments (Kotz et al., 2020).

### Predominant role of *Ruminococcaceae* and *Lachnospiraceae* in ruminal adaptation to a highly fermentable diet

Starch, the main non-structural carbohydrate included in our experimental diet, is degraded into glucose or glucose-1-phosphate in the rumen (Hoover and Miller, 1991; Mills et al., 1999). While the overall concentration of glucose tended to increase over the 6 days in our experiment, glucose-1-phosphate decreased. Previous studies have confirmed that increased concentrate intake results in a higher concentration of glucose and VFAs in the rumen, due to the augmented level of readily fermentable carbohydrates introduced with the diet (Ametaj et al., 2010; Saleem, 2012). The decreased concentration of glucose-1-phosphate found in our study could suggest a high metabolization rate through glycolysis, or possible accumulation in the form of glycogen (Lou et al., 1997; Mills et al., 1999; Hackmann, 2015). The main putative producers of glucose-1-phosphate in our study were identified as belonging to families *Ruminococcaceae* and *Lachnospiraceae* in digesta samples, through cellobiose phosphorylase (K00702), which catalyzes the reaction that transforms plant-derived cellobiose into D-glucose (Hamura et al., 2012). In fact, members of these two taxa used to be considered mainly fibrolytic bacteria (Bickhart and Weimer, 2018; Holman and Gzyl, 2019), but certainly their enzymatic apparatus allows them to metabolize more readily available sugars as well. A previous metagenomic study found several putative carbohydrate-active enzymes to be associated with both families in solid digesta (Wang et al., 2013), and in our experiment both families were confirmed as connected with the metabolism of carbohydrates by the network analysis, albeit with moderate correlations. In addition, random forest regression analysis identified several ASVs classified as belonging to families *Ruminococcaceae* and *Lachnospiraceae* to be highly descriptive of the adaptation of the microbiota composition in the two ruminal niches over the experimental days. Therefore, despite the limits and the predictive nature of our analyses, all approaches used strongly supported the major role of these two taxa, suggesting a highly adaptive metabolic potential and strong plasticity in response to progressive increases in dietary starch. Our predictive analysis also associated *Ruminococcaceae* and *Lachnospiraceae* with the variance of acetate, succinate, and phenylacetic acid, highlighting the need to better investigate the metabolic potential of these families and their adaptive roles in the ruminal environment.

### Diet shapes ruminal microbial metabolism and composition

Our analyses revealed that the main drivers of the shift in metabolites composition, noticeable over the last 2 days of experiment, were glucose, acetic acid, and butyric acid. Both PICRUSt and quantitative enrichment analysis appeared to confirm the importance of these metabolites, with the most abundant pathways being related with starch metabolism, for instance NONOXIPENT-PWY (pentose phosphate pathway (non-oxidative branch) I), PWY-5100 (pyruvate fermentation to acetate and lactate II), GLYCOGENSYNTH-PWY (glycogen biosynthesis I, from ADP-D-Glucose), and PWY-6737 (starch degradation V). According to the function prediction, families *Erysipelotrichaceae* and *Lachnospiraceae* synthesized most of the acetate in digesta samples. Deusch et al. (2017) suggested a role of *Erysipelotrichaceae* in the production of lactate in the rumen, while Chen et al. (2021) found a negative correlation between members of this family and acetate production. This is in contrast with our results, demonstrating the need to further investigate the metabolic capabilities of the *Erysipelotrichaceae* family.

Genera *Bacillus, Prevotella, Ruminobacter*, and *Selenomonas* were identified in literature as main microorganisms producing propionate in the rumen, expressing high levels of succinate-CoA synthetase (Wang et al., 2020). Although the same pathway was not identified among the most abundant groups in our experiment, succinic acid concentration tended to increase in parallel with propionate concentration. Furthermore, bacteria belonging to family *Prevotellaceae* (in particular *Prevotellaceae* NK3B31 group and *Prevotellaceae* UCG-001) and *Selenomonas* 1 were found to increase over the 6 days in PARL and digesta, confirming their importance in high-concentrate diet digestion in the rumen. While *Prevotella* was not associated with succinic acid production in PARL samples in our study, family *Veillonellaceae*, to which genus *Selenomonas* belongs, was indicated as one of the major putative synthesizers. However, *Prevotella* plays a main role in carbohydrate metabolism and their role as main functional group in planktonic microbiota in the production of butyrate during a high-concentrate feeding regime was also confirmed (Wirth et al., 2018; Wang et al., 2020).

### Microbial metabolism produces potentially harmful metabolites in response to the dietary change

Some microbial by-products could pose a risk for animal health when their concentration is increased and remains at high levels. The reshaping of ruminal microflora in high-concentrate feeding is often accompanied by increased production of potentially harmful compounds, such as lipopolysaccharide (LPS), lactic acid, and biogenic amines (Khafipour et al., 2009; Saleem, 2012). Increased concentrations of ethanolamine are a common finding when cows are fed high energy diets, and augmented epithelial turnover and bacterial cell lysis were indicated as possible causes (Saleem, 2012; Zhang et al., 2017).

Sedoheptulose 7-phosphate is part of the pentose phosphate pathways associated with carbohydrate metabolism, as well as a fundamental element for LPS synthesis in Gram-negative bacteria (Taylor et al., 2008). In PARL samples, *Prevotellaceae* and *Succinivibrionaceae*, both Gram-negative and having increased frequency in the first 4 days of experiment, were the two families more strongly associated with the variation of sedoheptulose 7-phosphate. It is possible that the decreased concentration of the metabolite was due to its utilization, through D-sedoheptulose-7-phosphate isomerase (K03271), for the formation of LPS for the outer membrane of the bacteria.

The abundance of known lactic acid producing groups, such as *Streptococcaceae, Pseudobutyrivibrio* and *Butyrivibrio* 2, significantly increased in the rumen only on day 6 (Mackie and Gilchrist, 1979; Hernandez et al., 2008). We found a positive correlation between lactic acid and *Streptococcus* in digesta samples, as well as with genera *Lachnobacterium* and *FD2005*, belonging to family *Lachnospiraceae*. Both have already been associated with SARA in goats (Chen et al., 2021), but the production of this potentially harmful metabolite is reported only for members of the genus *Lachnobacterium* (Whitford et al., 2001). Interestingly, our results indicated that families *Ruminococcaceae* and *Succinivibrionaceae*, while not being generally associated with lactate production, have the potential to produce lactate, and could play a role along with other microbes in the susceptibility of an animal to SARA.

### Effects of the PFA on microbiota of the GIT

In our study, the phytogenic addition resulted in a higher abundance of *Paludibacteriaceae, Campylobacteraceae*, and *Clostridiales* Family XIII in the fecal samples. These families are typically described as members of the core microbiota in a healthy GIT (Dong et al., 2016; Plaizier et al., 2017; Holman and Gzyl, 2019). Although some strains belonging to the latter two taxa are considered as pathogens, their shedding seems to be more associated with seasonality, husbandry, or individuality, rather than with families relative abundance in the feces (Sproston et al., 2011; Dong et al., 2016). Genus *Paludibacter*, on the other hand, has been previously described as negatively affected by the high amount of carbohydrates in the diet, suggesting a positive role played by the phytogenic additive in preserving a healthy microbiota.

The phytogenic addition resulted in higher alpha diversity indices, especially toward the end of the experiment. This is an interesting result, as it seems that high diversity is a key factor in microbiota resilience (Sommer et al., 2017). Albeit affecting only relatively small abundant taxa, as confirmed by the significant difference found only for unweighted UniFrac, the phytogenic additive demonstrated to have the potential to alter the microbial activity in the rumen and to preserve a diverse microbiota across the GIT. Spermine and spermidine were previously reported as increased due to high concentrate feeding, but the level of both amines in the rumen was successfully reduced with supplementation with a phytogenic additive in a previous study (Humer et al., 2018a). In our experiment, only spermidine concentration was reduced by the phytogenic additive. Although biogenic amines receive a lot of attention especially in food contamination, research has not yet completely elucidated how substances, such as thymol, menthol, or eugenol, impact this aspect of microbial metabolism (Naila et al., 2010; Özogul et al., 2015).

However, the modifications in bacterial composition and activity were not enough to cause major fluctuations in the production of the other metabolites over the experimental period. It is possible that these changes could be more pronounced in animals undergoing a more severe dietary disruption. Further research is necessary to investigate the mechanism of action of the phytogenic additive under conditions of increasing severity, in order to understand its potential toward mitigation of dysbiosis in animals fed concentrate-rich diets.

## Conclusion

Rapid diet changes and increasing amounts of starch are responsible for rapid shifts in microbiota composition and activity in cattle GIT. Microorganisms inhabiting the rumen and hindgut of dairy cattle demonstrated a high adaptation capacity, with the effects of perturbation of the ecosystems starting to be visible with inclusion of 50% concentrate in the diet. As expected, readily fermentable carbohydrates increased glucose, VFAs, and related metabolites and pathways both in the rumen and in feces, with bacteria belonging to families *Ruminococcaceae* and *Lachnospiraceae* showing a great plasticity and capacity to adapt rapidly to sudden dietary shifts. Supplementation with a PFA that includes menthol, thymol, and eugenol showed potential beneficial effects by increasing the diversity of the microbiota, despite affecting only relatively small abundant taxa. Lastly, our work contributes to better understanding of PARL: this lesser studied digesta fraction, whose microbiota is at the interface between solid and liquid rumen content, is highly important in the utilization of nutrients. Therefore, modifying its composition through the use of feed additives could be a target for the development of nutritional interventions to improve digestion and rumen health in cattle.

## Data availability statement

The datasets presented in this study can be found in online repositories. The names of the repository/repositories and accession number(s) can be found below: https://www.ncbi.nlm.nih.gov/, BioProject PRJNA769001.

## Ethics statement

The animal study was reviewed and approved by Institutional Ethics and Animal Welfare Committee of the University of Veterinary Medicine Vienna and the Austrian national authority according to the law for animal experiments (Protocol Number: BMBWF-68.205/0003-V/3b/2019).

## Author contributions

QZ leads the CD laboratory and acquired funding with RP, NR, HS-Z, and FB. QZ, RP, and NR designed the experiment. SR, RR-C, RP, and EC-L performed the experimental trial. SR and CP performed the DNA extractions. HS-Z carried out the metabolomics analyses. SR performed bioinformatics and statistical analyses, with the help of QZ, RP, and CP. SR wrote the manuscript, that was revised and approved by all authors. All authors contributed to the article and approved the submitted version.

## Funding

This work has been funded by the Christian Doppler Laboratory for Innovative Gut Health Concepts of Livestock, the Austrian Federal Ministry for Digital and Economic Affairs and the National Foundation for Research, Technology and Development.

## Conflict of interest

NR is employed at DSM, BIOMIN Research Center, a company that manufactures feed additives and financially supports the Christian Doppler Laboratory for Innovative Gut Health Concepts of Livestock. However, the authors declare that this had no impact on the analysis or interpretation of results.

The remaining authors declare that the research was conducted in the absence of any commercial or financial relationships that could be construed as a potential conflict of interest.

## Publisher's note

All claims expressed in this article are solely those of the authors and do not necessarily represent those of their affiliated organizations, or those of the publisher, the editors and the reviewers. Any product that may be evaluated in this article, or claim that may be made by its manufacturer, is not guaranteed or endorsed by the publisher.
