# Supplementary material for: Progressive microbial adaptation of the bovine rumen and hindgut in response to a step-wise increase in dietary starch and the influence of phytogenic supplementation
Source: Front Microbiol. 2022 Jul 22;13:920427. doi: 10.3389/fmicb.2022.920427 (PMC9354822; doi:10.3389/fmicb.2022.920427)
Supplement: Supplementary file 1 [file Data_Sheet_1.docx]

Supplementary Material

# Supplementary Data

**Solvents and reagents for rumen metabolomics analysis**

Solvents and reagents were of analytical grade or higher and obtained from VWR International GmbH (Vienna, Austria), Sigma-Aldrich (Vienna, Austria), and Merck (Darmstadt, Germany). Water was purified with an Arium® pro Ultrapure Lab Water System (Sartorius, Göttingen, Germany). Reference standards for all analytes were purchased from Sigma Aldrich (Vienna, Austria), VWR (Vienna, Austria), and Cayman Chemicals (Ann Arbor, Michigan, USA).

**Chromatographic and mass spectrometric conditions and quantification approaches**

Analytes were separated on a Dionex IonPac AS11-HC column (250 x 2 mm, 4 μm particle size, Thermo Scientific) protected by a Dionex IonPac AG11-HC guard column (50 x 2 mm, 4 μm) at 30 °C. After separation, potassium ions were exchanged against protons by a Dionex AERS 500, 2 mm suppressor operated at 76 mM. To assist ionization, methanol was added to the mobile phase after passing the suppressor at a flow rate of 0.06 ml/min. Mass spectrometric detection was carried out in full scan mode (m/z 50-750) at a resolution of 70,000 after electrospray ionization (ESI) in negative mode. The automatic gain control target was 1 x 106 ions and 200 ms was set as maximum injection time. Thermo Xcalibur software was used for instrument control and data analysis. Analytes were quantified on the basis of pure solvent calibration curves established between 0.1 and 9,000 ng/ml for all analytes. Disaccharides that were coeluting under the chosen conditions were quantified using sucrose as reference standard.

LC-MS/MS analysis was performed on an Agilent 1290 UHPLC system (Agilent Technologies, Waldbronn, Germany) coupled to a QTrap 6500+ mass spectrometer equipped with an IonDrive TurboV source (SCIEX, Foster City, CA, USA). Analytes were separated on a Kinetex C18 column (50×2.1 mm, 1.7 μm particle size, Phenomenex, Aschaffenburg, Germany) at a flowrate of 0.6 ml/min and at a temperature of 50 °C. The injection volume was 2 µl. Mass spectrometric detection was performed in positive electrospray ionization mode and selected reaction monitoring (SRM) was applied as scan type. The SRM parameters are given in Table S1, Additional file 2. The source parameters were as follows: source temperature 500 °C, ion spray voltage 5500 V, curtain gas 45 psi, ion source gas 1 60 psi and ion source gas 2 70 psi. Analyst software version 1.6.3 (SCIEX) was employed for instrument control and data analysis. Quantitative analysis was performed on the basis of linear or quadratic calibration curves in the range between 0.6 and 1,000 ng/ml of derivatized reference standard compounds in measurement solution.

**DNA extraction protocol**

DNA was extracted using DNeasy PowerSoil Kit (Qiagen, Germany) with additional pre-processing steps for mechanical and enzymatic lysis. For the solid digesta and feces, approximately 250 mg of sample were cut into small pieces and put into the bead beating tubes. For PARL, 800 µl of fluid were loaded into the tubes. The following steps were the same for all the matrices. After adding 60 μl of solution C1, samples were incubated for 5 min at 95 °C and then centrifuged at 10’000 × *g* for 2 minutes. The supernatant was kept on ice for later procession. A total of 100 μl of lysozyme (100 mg/ml, Sigma-Aldrich) and 10 μl of mutanolysin (2.5 U/μl, Sigma-Aldrich) were added to the pellet, and the samples were incubated at 37 °C for 30 min. The pellet was then incubated with 21.3 μl of Proteinase K (18.8 mg/ml, Sigma-Aldrich) for 1 hour at 37 °C before being homogenized using a bead beater (FastPrep-Homogenizer 24^TM^ 5G, MP Biomedicals, Santa Ana, CA). The lysate obtained was mixed to the supernatant from previous steps. Afterward, the extraction followed the manufacturer’s instructions. DNA was eluted with 100 μl of solution C6. The concentration of DNA extracted from each sample was measured with Qubit fluorometer (Qubit™ 4 Fluorometer, Thermo Fischer Scientific, USA).

# Supplementary Figures and Tables

## Supplementary Tables

**Supplementary Table S1.** Selected reaction monitoring (SRM) transitions for liquid chromatography - tandem mass spectrometric measurements. Quantification was based on linear or quadratic pure solvent calibration curves established between 0.0006 and 1 mg/L in measurement solution for all derivatized analytes. (quant = quantifier, qual = qualifier, CE = collision energy).

| **Analyte** | **Retention time (min)** | **Precursor ion (*m/z*)** | **Declustering potential (V)** | **Product ions (quant/qual, *m/z*)** | **CE (quant/qual, eV)** |
| --- | --- | --- | --- | --- | --- |
| Creatinine | 0.25 | 114.1 | 60 | 44.1/86.0 | 20/15 |
| Carnitine | 0.25 | 162.1 | 60 | 103.0/85.0 | 25/30 |
| Betaine | 0.26 | 118.1 | 70 | 58.0/59.0 | 35/25 |
| Creatine | 0.26 | 132.1 | 50 | 90.0/20.0 | 44/45 |
| Trigonelline | 0.26 | 138.0 | 95 | 92.0/94.0 | 30/35 |
| Taurine | 1.05 | 261.0 | 70 | 126.0/136.0 | 20/30 |
| Histamine | 1.12 | 247.0 | 70 | 154.0/95.0 | 18/35 |
| Carnosine | 1.13 | 362.2 | 70 | 207.0/269.0 | 25/20 |
| Ethanolamine | 1.73 | 197.1 | 70 | 136.0/62.0 | 25/18 |
| Sarcosine | 2.20 | 225.0 | 70 | 90.0/136.0 | 15/30 |
| β-Alanin | 2.41 | 225.0 | 70 | 90.0/136.0 | 15/30 |
| γ-Aminobutyric acid | 2.79 | 239.0 | 70 | 104.0/87.0 | 15/25 |
| β-Aminobutyric acid | 3.20 | 239.0 | 70 | 104.0/44.0 | 15/30 |
| 5-Aminovaleric acid | 3.49 | 253.0 | 70 | 100.0/94.0 | 23/22 |
| α-Aminobutyric acid | 3.79 | 239.0 | 70 | 104.0/58.0 | 15/30 |
| Pyrrolidine | 3.82 | 207.0 | 70 | 114.0/72.0 | 20/40 |
| Dopamine | 3.87 | 289.2 | 70 | 137.0/91.0 | 25/52 |
| Serotonin | 4.06 | 312.3 | 70 | 160.0/115.1 | 30/65 |
| Kynurenine | 4.43 | 344.2 | 70 | 192.0/146.0 | 20/30 |
| Putrescine | 4.55 | 359.1 | 70 | 266.0/114.0 | 15/35 |
| ^13^C-Putrescin | 4.55 | 363.1 | 70 | 270.1/118.0 | 15/35 |
| Cadaverin | 4.65 | 373.1 | 70 | 280.0/128.0 | 20/35 |
| 2-Phenylethylamine | 4.73 | 257.2 | 70 | 105.0/94.0 | 30/20 |
| Spermidine | 4.81 | 551.2 | 70 | 193.1/416.2 | 40/20 |
| Spermine | 4.92 | 743.3 | 70 | 473.0/193.1 | 25/60 |

**Supplementary Table S2.** Alpha diversity matrices calculated for rumen samples (solid digesta and PARL^1^). Values are presented per each day of adaptation to a high concentrate diet (from 10% to 60% concentrate) for control (CON) and treatment (PHY) group. *P*-values are presented for the effect of the adaptation day (Day), for the phytogenic supplementation (PHY) and for their interaction (Day*PHY).

| **DIGESTA** | **Day 1** | | **Day 2** | | **Day 3** | | **Day 4** | | **Day 5** | | **Day 6** | |  | | ***P*-values** | | |
| --- | --- | --- | --- | --- | --- | --- | --- | --- | --- | --- | --- | --- | --- | --- | --- | --- | --- |
|  | **CON** | **PHY** | **CON** | **PHY** | **CON** | **PHY** | **CON** | **PHY** | **CON** | **PHY** | **CON** | **PHY** | **SEM^3^** | **Day** | **PHY** | **Day*PHY** |  |
| ACE^2^ | 2425 | 2385 | 2571 | 2378 | 2362 | 2573 | 2192 | 2405 | 1966^a^ | 2258^a^ | 1490^b^ | 1793^b^ | 129.9 | < 0.01 | 0.09 | 0.28 |  |
| Chao1 | 2353 | 2315 | 2497 | 2307 | 2291 | 2499 | 2126 | 2334 | 1907^a^ | 2195^a^ | 1448^b^ | 1741^b^ | 126.7 | < 0.01 | 0.09 | 0.28 |  |
| Faith´s Phylogenetic Diversity | 112.6 | 112.7 | 115.5 | 110.7 | 109.6 | 115.8 | 101.7 | 110.2 | 95.6^a^ | 105.2^a^ | 78.04^b^ | 88.85^b^ | 4.28 | < 0.01 | 0.03 | 0.27 |  |
| Shannon | 10.03 | 10.07 | 10.10 | 10.03 | 9.93 | 10.04 | 9.74 | 9.95 | 9.60^a^ | 9.72^a^ | 8.97^b^ | 9.24^b^ | 0.10 | < 0.01 | 0.11 | 0.61 |  |
| **PARL^1^** | **Day 1** | | **Day 2** | | **Day 3** | | **Day 4** | | **Day 5** | | **Day 6** | |  | | ***P*-values** | | |
|  | **CON** | **PHY** | **CON** | **PHY** | **CON** | **PHY** | **CON** | **PHY** | **CON** | **PHY** | **CON** | **PHY** | **SEM^3^** | **Day** | **PHY** | **Day*PHY** |  |
| ACE^2^ | 2081 | 2078 | 2055^x^ | 1788^x^ | 2204^y^ | 2311^y^ | 1932^x^ | 1934^x^ | 1495^y^ | 1765^y^ | 1643 | 1617 | 129.9 | < 0.01 | 0.85 | 0.34 |  |
| Chao1 | 2016 | 2014 | 1994^x^ | 1732^x^ | 2134^y^ | 2240^y^ | 1872^x^ | 1872^x^ | 1450^y^ | 1711^y^ | 1596 | 1566 | 126.4 | < 0.01 | 0.86 | 0.34 |  |
| Faith´s Phylogenetic Diversity | 90.86 | 90.95 | 91.51 | 81.33 | 92.43^a^ | 97.22^a^ | 84.60^b^ | 85.48^b^ | 69.64^a^ | 78.25^a^ | 74.56 | 73.67 | 3.67 | < 0.01 | 0.81 | 0.14 |  |
| Shannon | 9.75 | 9.70 | 9.70 | 9.50 | 9.65^a^ | 9.73^a^ | 9.35^b^ | 9.31^b^ | 8.62^a^ | 9.04^a^ | 8.73 | 8.88 | 0.14 | < 0.01 | 0.45 | 0.12 |  |

^1^ Particle associated rumen liquid.

^2^ Abundance-based coverage estimator.

^3^ The largest standard error of the mean.

^a,b^ Values with different superscripts indicate a significant difference (*P* ≤ 0.05) between consecutive days.

^x,y^ Values with different superscripts indicate a tendency for difference (0.05 < *P* ≤ 0.10) between consecutive days.

**Supplementary Table S3.** Alpha diversity matrices calculated for fecal samples. Values are presented per each day of adaptation to a high concentrate diet (from 10% to 60% concentrate) for control (CON) and treatment (PHY) group. Fecal samples were collected for an additional seventh day. *P*-values are presented for the effect of the adaptation day (Day), for the phytogenic supplementation (PHY) and for their interaction (Day*PHY).

|  | **Day 1** | | **Day 2** | | **Day 3** | | **Day 4** | | **Day 5** | | **Day 6** | | **Day 7** | |  | | ***P*-values** | |
| --- | --- | --- | --- | --- | --- | --- | --- | --- | --- | --- | --- | --- | --- | --- | --- | --- | --- | --- |
|  | **CON** | **PHY** | **CON** | **PHY** | **CON** | **PHY** | **CON** | **PHY** | **CON** | **PHY** | **CON** | **PHY** | **CON** | **PHY** | **SEM^2^** | **Day** | **PHY** | **Day*PHY** |
| ACE^1^ | 823.7 | 806.6 | 715.8 | 671.9 | 776.7 | 901.0 | 1022 | 681.3 | 973.8 | 833.2 | 715.9 | 670.0 | 687.8 | 637.5 | 100.1 | 0.03 | 0.08 | 0.31 |
| Chao1 | 795.4 | 776.7 | 690.0 | 647.5 | 748.0 | 869.1 | 988.4 | 657.9 | 939.8 | 805.9 | 693.6 | 647.6 | 666.4 | 617.4 | 96.6 | 0.03 | 0.08 | 0.30 |
| Faith´s Phylogenetic Diversity | 37.16 | 36.70 | 32.86 | 32.38 | 34.58 | 39.60 | 40.70 | 32.27 | 39.60 | 36.59 | 32.36 | 31.19 | 31.18 | 28.98 | 2.89 | 0.01 | 0.20 | 0.36 |
| Shannon | 8.51 | 8.48 | 8.32 | 8.15 | 8.23 | 8.46 | 8.33 | 8.19 | 8.28 | 8.33 | 7.96 | 8.07 | 7.91 | 7.90 | 0.18 | 0.01 | 0.95 | 0.88 |

^1^ Abundance-based coverage estimator.

^2^ The largest standard error of the mean.

**Supplementary Table S4.** Metabolites measured in rumen fluid over the six days of experiment. Concentration is given in µg/ml per each day for the control (CON) and the treatment (PHY) groups. *P*-values are presented for the effect of the adaptation day (Day), for the phytogenic supplementation (PHY) and for their interaction (Day*PHY). (SEM = largest standard error of the mean).

|  | **Day 1** | | **Day 2** | | **Day 3** | | **Day 4** | | **Day 5** | | **Day 6** | |  | ***P*-values** | | |
| --- | --- | --- | --- | --- | --- | --- | --- | --- | --- | --- | --- | --- | --- | --- | --- | --- |
|  | **CON** | **PHY** | **CON** | **PHY** | **CON** | **PHY** | **CON** | **PHY** | **CON** | **PHY** | **CON** | **PHY** | **SEM** | **Day** | **PHY** | **Day*PHY** |
| Ribose | 20.4 | 18.8 | 34.7 | 26.2 | 35.1^a^ | 32.3^a^ | 61.1^b^ | 58.9^b^ | 107^c^ | 57.1^d^ | 138 | 122 | 0.42 | < 0.01 | 0.06 | 0.08 |
| Glyceric acid | 2.01 | 2.03 | 2.02^a^ | 2.04^a^ | 1.38^b^ | 1.37^b^ | 1.72 | 1.84 | 2.05 | 1.70 | 1.47 | 1.52 | 0.24 | < 0.01 | 0.87 | 0.81 |
| 3-Hydroxybutyric acid | 3.62^a^ | 3.52^a^ | 2.37^b^ | 2.41^b^ | 1.26^a^ | 1.16^a^ | 1.00^a^ | 0.61^a^ | 3.55^b^ | 2.42^b^ | 4.02^c^ | 1.70^d^ | 0.50 | < 0.01 | 0.23 | 0.01 |
| Glycolic acid | 8.09 | 6.52 | 6.26 | 7.37 | 5.33 | 4.77 | 6.72 | 4.39 | 4.95 | 6.21 | 5.53 | 5.79 | 1.00 | 0.10 | 0.59 | 0.30 |
| 2-Methylbutyric acid | 102 | 86.3 | 85.6^a^ | 90.5^a^ | 59.5^b^ | 61.0^b^ | 57.9 | 68.8 | 52.2 | 61.7 | 43.1 | 51.5 | 11.3 | < 0.01 | 0.81 | 0.36 |
| D-Galacturonic acid | 0.28 | 2.13 | 2.19 | 2.65 | 2.73 | 3.08 | 1.23 | 1.34 | 4.99 | 5.69 | 7.70 | 4.02 | 1.39 | 0.01 | 0.97 | 0.22 |
| 2-Ethylbutyric acid | 3.44^a^ | 3.34^a^ | 2.51^b^ | 2.44^b^ | 1.49^a,x^ | 1.36^a,x^ | 1.05^b,y^ | 0.96^b,y^ | 3.40^a^ | 2.93^a^ | 2.14^b^ | 2.11^b^ | 0.24 | < 0.01 | 0.34 | 0.87 |
| Hexanoic acid | 127 | 107 | 145^a^ | 121^a^ | 106^b^ | 95.7^b^ | 115 | 127 | 132 | 124 | 99.8 | 106 | 12.5 | 0.01 | 0.43 | 0.45 |
| Galactose-1-phosphate | 1.93^a^ | 2.24^a^ | 1.23^b^ | 1.45^b^ | 0.75 | 0.73 | 1.01^a^ | 0.45^a^ | 2.53^b^ | 2.31^b^ | 1.28^v^ | 2.41^z^ | 0.54 | < 0.01 | 0.44 | 0.08 |
| Phenylacetic acid | 72.4 | 65.0 | 66.6^a^ | 64.7^a^ | 51.3^b^ | 48.8^b^ | 44.5 | 45.4 | 48.9 | 41.4 | 41.5 | 38.4 | 5.00 | < 0.01 | 0.29 | 0.91 |
| Succinic acid | 7.42 | 8.78 | 6.76 | 8.17 | 12.3 | 6.85 | 7.68 | 7.96 | 10.9^a^ | 6.86^a^ | 19.5^b^ | 20.9^b^ | 1.33 | < 0.01 | 0.70 | 0.34 |
| D-Glucose-6-phosphate | 4.64^a^ | 4.77^a^ | 3.31^b^ | 3.49^b^ | 2.64^a^ | 2.65^a^ | 2.15^a^ | 2.56^a^ | 3.86^b^ | 3.96^b^ | 2.45^a^ | 2.65^a^ | 0.27 | < 0.01 | 0.39 | 0.91 |
| Benzoic acid | 6.94 | 6.47 | 7.16^a^ | 6.99^a^ | 4.84^b^ | 3.82^b^ | 2.98 | 3.18 | 4.03 | 4.30 | 2.86 | 2.62 | 1.93 | < 0.01 | 0.62 | 0.96 |
| D-Mannose-6-phosphate | 2.86^a^ | 3.05^a^ | 1.92^b^ | 1.87^b^ | 1.15^a^ | 1.18^a^ | 0.61^b^ | 0.46^b^ | 2.64^a^ | 2.72^a^ | 1.68^b^ | 1.71^b^ | 0.32 | < 0.01 | 0.92 | 0.76 |
| Disaccharides | 31.7 | 25.95 | 47.3 | 37.6 | 48.0^x^ | 42.4^x^ | 79.4^y^ | 77.5^y^ | 180^c^ | 50.8^d^ | 116 | 87.2 | 1.51 | < 0.01 | 0.39 | 0.13 |
| Glucose | 128 | 98.2 | 202 | 162 | 222^a^ | 178^a^ | 428^b^ | 407^b^ | 985 | 376 | 665 | 1067 | 1.34 | < 0.01 | 0.30 | 0.13 |
| Pyroglutamate | 3.62 | 3.49 | 3.14 | 3.42 | 3.38 | 2.34 | 2.53 | 2.39 | 3.06 | 3.13 | 2.80 | 3.39 | 24.1 | 0.10 | 0.64 | 0.42 |
| Glucose-1-phosphate | 3.03^a^ | 3.09^a^ | 2.52^b,x^ | 2.58^b,x^ | 2.09^y^ | 2.33^y^ | 2.00^a^ | 2.05^a^ | 2.71^b^ | 3.05^b^ | 2.64^a^ | 2.21^a^ | 0.19 | < 0.01 | 0.69 | 0.14 |
| D-Sedoheptulose-7-phosphate | 3.09^a^ | 3.16^a^ | 2.22^b^ | 2.20^b^ | 1.87^a^ | 1.77^a^ | 1.64^a^ | 1.79^a^ | 2.70^b^ | 2.79^b^ | 2.29^a^ | 2.06^a^ | 1.08 | < 0.01 | 0.94 | 0.58 |
| 3-Phenylpropionic acid | 116^a^ | 105^a^ | 135^b^ | 123^b^ | 118^a^ | 110^a^ | 123^a^ | 115^a^ | 104^b^ | 98.4^b^ | 104 | 9078 | 8.28 | < 0.01 | 0.31 | 0.97 |
| 3-(3-Hydroxyphenyl) propionic acid | 4.88 | 4.25 | 7.35 | 6.61 | 4.29 | 3.33 | 3.23 | 3.06 | 6.45 | 4.24 | 7.37 | 4.30 | 1.31 | 0.01 | 0.09 | 0.87 |

^a,b^ Values with different superscripts indicate a significant difference (*P* ≤ 0.05) between consecutive days.

^x,y^ Values with different superscripts indicate a tendency for difference (0.05 < *P* ≤ 0.10) between consecutive days.

^c,d^ Values with different superscripts indicate a significant difference (*P* ≤ 0.05) between treatment (PHY) and control (CON).

^v,z^ Values with different superscripts indicate a tendency for difference (0.05 < *P* ≤ 0.10) between treatment (PHY) and control (CON).

**Supplementary Table S5.** Model results of MIMOSA2 software for solid digesta samples. Results are presented only for the metabolites considered significant (*P* ≤ 0.05 and FDR < 0.1). (Synth/Deg Species = number of identified synthesizing/degrading taxa, Synth/Deg Genes = synthesizing/degrading genes identified in the dataset.

| Digesta |  |  |  |  |  |  |  |  |  |  |  |
| --- | --- | --- | --- | --- | --- | --- | --- | --- | --- | --- | --- |
| KEGG ID | **Metabolite** | **Intercept** | **Slope** | **R^2^** | ***P*-value** | **R^2^  Adjusted** | **FDR** | **Synth Species** | **Deg Species** | **Synth Genes** | **Deg Genes** |
| C00033 | Acetate | 10518.62 | -20.254 | 0.035 | 0.007 | 0.068 | 0.018 | 1166 | 0 | K01512 K01067 K00156 K00467 |  |
| C00092 | D-Glucose 6-phosphate | 5.46 | -0.009 | 0.074 | < 0.001 | 0.145 | < 0.001 | 1270 | 0 | K01193 K01222 K01223 K01232 K01226 |  |
| C00256 | (R)-Lactate | 42.12 | -0.595 | 0.020 | < 0.001 | 0.169 | < 0.001 | 645 | 31 | K01069 | K00102 |
| C00275 | D-Mannose 6-phosphate | 4.13 | -0.015 | 0.043 | 0.004 | 0.075 | 0.014 | 770 | 0 | K02793 K02794 K02795 K02796 K00844 |  |
| C07086 | Phenylacetic acid | 106.58 | -0.491 | 0.050 | 0.001 | 0.093 | 0.006 | 670 | 0 | K02614 |  |
| C00315 | Spermidine | -5.90 | 0.036 | 0.063 | < 0.001 | 0.113 | 0.005 | 1926 | 15 | K00797 K13747 | K01917 K00316 |
| C00431 | 5-Aminovaleric acid | 41.71 | 13.895 | 0.032 | 0.004 | 0.075 | 0.017 | 37 | 0 | K10793 K10794 |  |
| C00750 | Spermine | -0.84 | 0.008 | 0.026 | 0.002 | 0.085 | 0.014 | 1336 | 0 | K00797 |  |

**Supplementary Table S6.** Model results of MIMOSA2 software for particle associated rumen liquid (PARL) samples. Results are presented only for the metabolites considered significant (*P* ≤ 0.05 and FDR < 0.1). (Synth/Deg Species = number of identified synthesizing/degrading taxa, Synth/Deg Genes = synthesizing/degrading genes identified in the dataset.

| PARL |  |  |  |  |  |  |  |  |  |  |  |
| --- | --- | --- | --- | --- | --- | --- | --- | --- | --- | --- | --- |
| KEGG ID | **Metabolite** | **Intercept** | **Slope** | **R2** | ***P*-value** | **R2 Adjusted** | **FDR** | **Synth Species** | **Deg Species** | **Synth Genes** | **Deg Genes** |
| C00042 | Succinate | 14.42 | -0.188 | 0.012 | 0.036 | 0.042 | 0.079 | 267 | 0 | K01739 K01637 K10764 |  |
| C00092 | D-Glucose 6-phosphate | 4.30 | -0.004 | 0.021 | 0.036 | 0.042 | 0.079 | 1340 | 0 | K01193 K01222 K01223 K01232 K01226 |  |
| C00256 | (R)-Lactate | 31.18 | -0.377 | 0.017 | < 0.001 | 0.157 | < 0.001 | 682 | 44 | K01069 | K00102 |
| C05382 | Sedoheptulose 7-phosphate | 3.00 | 0.022 | 0.023 | 0.025 | 0.048 | 0.079 | 0 | 298 |  | K03271 |
| C00099 | Beta-alanine | -0.23 | 0.031 | 0.018 | 0.047 | 0.038 | 0.081 | 1054 | 23 | K00128 | K09722 |
| C00134 | Putrescine | 2.93 | -0.080 | 0.013 | 0.020 | 0.051 | 0.041 | 1792 | 2027 | K01480 K12251 | K00797 K00657 K12256 K09470 |
| C00315 | Spermidine | -0.39 | 0.017 | 0.047 | 0.001 | 0.096 | 0.004 | 1930 | 17 | K00797 K13747 | K01917 K00316 |
| C00431 | 5-Aminovaleric acid | 39.22 | 8.123 | 0.039 | 0.001 | 0.097 | 0.004 | 48 | 0 | K10793 K10794 |  |
| C00750 | Spermine | -0.70 | 0.006 | 0.048 | < 0.001 | 0.148 | < 0.001 | 1394 | 0 | K00797 |  |
| C01672 | Cadaverine | 63.25 | 0.162 | 0.031 | 0.004 | 0.079 | 0.009 | 0 | 1394 |  | K00797 |
| C05332 | Phenylethylamine | 2.51 | -1.372 | 0.078 | < 0.001 | 0.167 | < 0.001 | 3 | 0 | K01593 |  |

**Supplementary** **Table S7.** Most important ASVs identified with random forest regression. Random forest regression was performed with “longitudinal maturity-index” function from q2-longitudinal plugin in QIIME 2. Results are presented per each analyzed matrix (digesta, particle associated rumen liquid (PARL) and feces). (Importance = importance scores as calculated by the function; Relative frequency = relative frequency in % across the whole dataset of a specific matrix).

|  |  |  |  |  | **Classification** | | |
| --- | --- | --- | --- | --- | --- | --- | --- |
| **ASV** | **Feature** | **Importance** | **Sample type** | **Relative frequency (%)** | **Phylum** | **Family** | **Genus** |
| ASV_1 | 98aae627ebf61c7b8916102643ebca25 | 0.201 | Digesta | 0.17 | *Firmicutes* | *Ruminococcaceae* | *Papillibacter* |
| ASV_2 | 52152536c1a92c51915d30b0877cb9fc | 0.069 | Digesta | 0.44 | *Firmicutes* | *Lachnospiraceae* | *Lachnospiraceae* XPB1014 group |
| ASV_3 | c09c8e0b9aa726b12ec31e66b2856f3d | 0.048 | Digesta | 0.50 | *Firmicutes* | Family XIII | Family XIII AD3011 group |
| ASV_4 | 1c3b8c9fee1dd22471f000ad8203a19d | 0.047 | Digesta | 0.03 | *Firmicutes* | Family XIII | [*Eubacterium*] brachy group |
| ASV_5 | 507c71b6d2d93f01469705fb6b389dbe | 0.032 | Digesta | 0.19 | *Firmicutes* | *Lachnospiraceae* | *Acetitomaculum* |
| ASV_6 | b21c8040fb9243610114f59bb5b9973d | 0.030 | Digesta | 0.09 | *Actinobacteria* | *Atopobiaceae* | *Atopobium* |
| ASV_7 | 9ff7572e7c109aa18351e457fcbd126b | 0.017 | Digesta | 0.01 | *Firmicutes* | *Christensenellaceae* |  |
| ASV_8 | 73342140a5c2ea05a71d2993ad093ecb | 0.016 | Digesta | 0.07 | *Firmicutes* | *Lachnospiraceae* | *Lachnoclostridium* |
| ASV_9 | d864cc97412dde9c8e66d54a5e0c12af | 0.016 | Digesta | 0.04 | *Firmicutes* | *Lachnospiraceae* | *Lachnospiraceae* ND3007 group |
| ASV_10 | a9e241c1ad23939c707499e2eb74d04c | 0.015 | Digesta | 0.16 | *Firmicutes* | *Lachnospiraceae* | *Lachnospiraceae* NK3A20 group |
| ASV_11 | eb29e7fa6967348f17ed63affef235c3 | 0.015 | Digesta | 0.02 | *Firmicutes* | *Lachnospiraceae* |  |
| ASV_12 | de0a98520625c5b1c70d6071b13aaef2 | 0.015 | Digesta | 0.01 | *Firmicutes* | *Christensenellaceae* | *Christensenellaceae* R-7 group |
| ASV_13 | 2ce76353cdbcacd58b51450dd77775b3 | 0.014 | Digesta | 0.08 | *Bacteroidetes* | *Prevotellaceae* | *Prevotella* 1 |
| ASV_14 | 5d40e46f30e46586c8aba520249cdd83 | 0.011 | Digesta | 0.16 | *Firmicutes* | *Lachnospiraceae* | *Lachnospiraceae* NK3A20 group |
| ASV_15 | abb711d62504a05cc2fd164779378d34 | 0.009 | Digesta | 0.57 | *Firmicutes* | *Ruminococcaceae* | *Ruminococcaceae* NK4A214 group |
| ASV_16 | 4730173ecf9176991efc17f7fef33b02 | 0.009 | Digesta | 0.04 | *Firmicutes* | *Lachnospiraceae* | *Lachnospiraceae* AC2044 group |
| ASV_17 | 85b34843774ce9a8520b2a07e03f67e0 | 0.009 | Digesta | 0.10 | *Firmicutes* | *Lachnospiraceae* | *Roseburia* |
| ASV_18 | 08b9fe79ed79acb4ef3bb58f4850d707 | 0.008 | Digesta | 0.04 | *Firmicutes* | *Ruminococcaceae* |  |
| ASV_19 | 440994cff31b8a7b27e88399af03bf55 | 0.008 | Digesta | 0.04 | *Actinobacteria* | *Eggerthellaceae* | DNF00809 |
| ASV_20 | 088307ceba2fc4cd9f1453facdca65e3 | 0.008 | Digesta | 0.14 | *Firmicutes* | *Lachnospiraceae* | probable genus 10 |
| ASV_21 | 3fe64e4208815c59dd93de2d2fdaf481 | 0.008 | Digesta | 0.03 | *Firmicutes* | *Ruminococcaceae* |  |
| ASV_22 | 64723344560a40480cf105689aeeade8 | 0.008 | Digesta | 0.02 | *Firmicutes* | *Lachnospiraceae* | *Lachnospiraceae* NK3A20 group |
| ASV_23 | 9d49049df883d65b0105b0fe8d679c1b | 0.008 | Digesta | 0.25 | *Firmicutes* | Family XIII | Family XIII AD3011 group |
| ASV_24 | 54605fee1061dd852a801ca1d8d59e20 | 0.008 | Digesta | 0.04 | *Firmicutes* | *Christensenellaceae* | *Christensenellaceae* R-7 group |
| ASV_25 | 4ece7f1e34464ee5ac34630987a2bce4 | 0.008 | Digesta | 0.14 | *Firmicutes* | *Ruminococcaceae* | *Ruminococcaceae* UCG-011 |
| ASV_26 | 103002cc99f92ef5fba9e8b08ed1b8ad | 0.008 | Digesta | 0.04 | *Bacteroidetes* | *Bacteroidales* BS11 gut group | uncultured rumen bacterium |
| ASV_27 | 29ad4488aff3ceee157ae194d4d411b9 | 0.008 | Digesta | 0.03 | *Firmicutes* | *Ruminococcaceae* | [*Eubacterium*] coprostanoligenes group |
| ASV_28 | 26a8ebc9c055fae1d7a9efb7dfa19345 | 0.008 | Digesta | 0.01 | *Bacteroidetes* | CAP-aah99b04 | uncultured bacterium |
| ASV_29 | a27e72f6b555ba81476ffdf81f75de19 | 0.008 | Digesta | 0.07 | *Firmicutes* | *Lachnospiraceae* | *Lachnospiraceae* NK3A20 group |
| ASV_30 | 99e0868703df5654f58da718ef080985 | 0.008 | Digesta | 0.06 | *Firmicutes* | Family XIII | Family XIII AD3011 group |
| ASV_31 | 3819979c3048dfb0d6d1bd7ffe33ed8e | 0.008 | Digesta | 0.02 | *Firmicutes* | *Lachnospiraceae* | *Lachnospiraceae* NK4A136 group |
| ASV_32 | e531c971bb635a2ae56ac8550caf4137 | 0.008 | Digesta | 0.05 | *Firmicutes* | *Ruminococcaceae* | *Ruminococcaceae* UCG-014 |
| ASV_33 | 7dcd94645f8e9f54f1872833e47d18ba | 0.008 | Digesta | 0.02 | *Firmicutes* | *Ruminococcaceae* | *Ruminococcaceae* NK4A214 group |
| ASV_34 | 3e247aaf42f4f27498e6c8bffebffe38 | 0.007 | Digesta | 0.04 | *Firmicutes* | *Lachnospiraceae* | *Oribacterium* |
| ASV_35 | a87d45a720a51699b1202a06e7d8f6fc | 0.007 | Digesta | 0.02 | *Bacteroidetes* | F082 | uncultured rumen bacterium |
| ASV_36 | b894416db2a0d81d7405817049b83647 | 0.007 | Digesta | 0.02 | *Firmicutes* | *Lachnospiraceae* | *Lachnoclostridium* 10 |
| ASV_37 | c8a20b7e52fbabcf298fa471def6cd33 | 0.007 | Digesta | 0.02 | *Firmicutes* | *Lachnospiraceae* | *Blautia* |
| ASV_38 | 52fd67f18492d5a07bc11511e29f9096 | 0.007 | Digesta | 0.44 | *Firmicutes* | *Lachnospiraceae* | *Lachnospiraceae* NK3A20 group |
| ASV_39 | 19d26e88d18368dda0376f1c62da04df | 0.007 | Digesta | 0.07 | *Firmicutes* | *Ruminococcaceae* | *Ruminococcaceae* UCG-005 |
| ASV_40 | e64323235df7295f0e0054c371b927fd | 0.007 | Digesta | 0.02 | *Actinobacteria* | uncultured | uncultured *Coriobacteriaceae* bacterium |
| ASV_41 | b16f901de0bbefd2155d9c434e178bf7 | 0.007 | Digesta | 0.07 | *Bacteroidetes* | F082 | *Bacteroidales* bacterium Bact_22 |
| ASV_42 | a25c29c7ad1749d56bd47ad695c65cec | 0.007 | Digesta | 0.08 | *Firmicutes* | *Lachnospiraceae* | Uncultured |
| ASV_43 | ddb9750a9a3255362963f5347ac98352 | 0.007 | Digesta | 0.03 | *Bacteroidetes* | *Paludibacteraceae* | Uncultured |
| ASV_44 | 2e1b2176b05a83910483a419fe195b1c | 0.007 | Digesta | 0.04 | *Bacteroidetes* | *Rikenellaceae* | *Rikenellaceae* RC9 gut group |
| ASV_45 | 59ba4f126c09944012a362ae7f110e3d | 0.006 | Digesta | 0.17 | *Firmicutes* | *Ruminococcaceae* | *Ruminococcus* 1 |
| ASV_46 | 4e9f24f7ede1446ce6c2d08bff099d48 | 0.004 | Digesta | 0.03 | *Firmicutes* | *Lachnospiraceae* |  |
| ASV_47 | 5fe7ab57a4d5dfbbc3647156cb07ba81 | 0.004 | Digesta | 0.03 | *Kiritimatiellaeota* | uncultured rumen bacterium | uncultured rumen bacterium |
| ASV_48 | 3a865661633a2266f3551524bb097582 | 0.004 | Digesta | 0.04 | *Firmicutes* | *Ruminococcaceae* | *Ruminococcaceae* UCG-010 |
| ASV_49 | 04a96f29644c3aecbdea41f83c880158 | 0.004 | Digesta | 0.02 | *Firmicutes* | *Lachnospiraceae* |  |
| ASV_50 | aa44c9a444b29bae0f900ba9b9abf766 | 0.003 | Digesta | 0.04 | *Kiritimatiellaeota* | uncultured rumen bacterium | uncultured rumen bacterium |
| ASV_51 | bfc80f8a30653196a7fcffc42bb8dde9 | 0.120 | PARL | 0.57 | *Firmicutes* | *Lachnospiraceae* | *Acetitomaculum* |
| ASV_29 | a27e72f6b555ba81476ffdf81f75de19 | 0.108 | PARL | 0.08 | *Firmicutes* | *Lachnospiraceae* | *Lachnospiraceae* NK3A20 group |
| ASV_52 | 9a407e0a8ed2260416f5ac18699bab61 | 0.045 | PARL | 0.02 | *Firmicutes* | *Ruminococcaceae* | *Ruminococcaceae* V9D2013 group |
| ASV_14 | 5d40e46f30e46586c8aba520249cdd83 | 0.045 | PARL | 0.15 | *Firmicutes* | *Lachnospiraceae* | *Lachnospiraceae* NK3A20 group |
| ASV_53 | 426a1b0c36b4ccd4e5d6bde17ffd5164 | 0.031 | PARL | 0.06 | *Spirochaetes* | *Spirochaetaceae* | *Treponema* 2 |
| ASV_54 | cbd8a3f53d76e6b96aca08c9852eb80c | 0.027 | PARL | 0.41 | *Firmicutes* | *Lachnospiraceae* | *Lachnospiraceae* NK3A20 group |
| ASV_55 | 107fb2e843fe3a14ca507a860fc33595 | 0.024 | PARL | 0.03 | *Firmicutes* | *Lachnospiraceae* | *Lachnospiraceae* AC2044 group |
| ASV_56 | 8fb22b227c2052dc0657e55d5b1ae1c9 | 0.022 | PARL | 0.03 | *Bacteroidetes* | *Prevotellaceae* | *Prevotella* 1 |
| ASV_57 | c6c0615dd9dcdcb51387ab1cfc51e12b | 0.022 | PARL | 0.02 | *Firmicutes* | *Christensenellaceae* | *Christensenellaceae* R-7 group |
| ASV_58 | 46cbae39602d3b1dc49e700a2a5e77cf | 0.021 | PARL | 0.05 | *Firmicutes* | *Lachnospiraceae* | *Tyzzerella* 3 |
| ASV_59 | 59a7758e414664c2ed49a7a4ab0b56e5 | 0.021 | PARL | 0.01 | *Firmicutes* | *Ruminococcaceae* | [*Eubacterium*] coprostanoligenes group |
| ASV_60 | 5a3a39fba3f8ffbee4f7100df6ae6118 | 0.021 | PARL | 0.04 | *Tenericutes* | *Anaeroplasmataceae* | *Anaeroplasma* |
| ASV_61 | a57bbc4cc4df4f2e305c3fedb7207928 | 0.017 | PARL | 0.06 | *Firmicutes* | *Lachnospiraceae* | *Lachnospiraceae* NK3A20 group |
| ASV_62 | 45999d73336c630ba8c1a5c91e014600 | 0.016 | PARL | 0.04 | *Bacteroidetes* | *Bacteroidales* RF16 group | uncultured bacterium |
| ASV_63 | bce7a04b72049ec48f51730be6a1fe5c | 0.016 | PARL | 0.08 | *Patescibacteria* | *Saccharimonadaceae* | *Candidatus* *Saccharimonas* |
| ASV_64 | 320a8b20b807993b001a49afc285bc10 | 0.015 | PARL | 0.09 | *Firmicutes* | *Lachnospiraceae* | *Butyrivibrio* 2 |
| ASV_65 | 25be18ced22201f667837c66621bb978 | 0.014 | PARL | 0.04 | *Firmicutes* | *Lachnospiraceae* | *Anaerosporobacter* |
| ASV_66 | eec48f119a432da7482270cce4a5a370 | 0.014 | PARL | 0.00 | *Firmicutes* | *Lachnospiraceae* | Butyrivibrio 2 |
| ASV_67 | f3f0d4ebefc7836c5b501c4ea4de051a | 0.012 | PARL | 0.07 | *Firmicutes* | *Leuconostocaceae* | *Weissella* |
| ASV_68 | c064e112376141deaebde982c400db42 | 0.010 | PARL | 0.08 | *Firmicutes* | *Lachnospiraceae* | *Lachnospiraceae* XPB1014 group |
| ASV_69 | 0331d5c8685a17220fbab29ddf6ceda8 | 0.008 | PARL | 0.17 | *Firmicutes* | *Lachnospiraceae* | [*Eubacterium*] hallii group |
| ASV_70 | 4afe6a25ea9a1591dbd654e4e3a8e770 | 0.008 | PARL | 0.01 | *Firmicutes* | *Ruminococcaceae* | *Ruminococcaceae* UCG-014 |
| ASV_71 | fb0195d6ae7e4148696cd138d75490a5 | 0.008 | PARL | 0.07 | *Kiritimatiellaeota* | uncultured rumen bacterium | uncultured rumen bacterium |
| ASV_72 | d20d5f21c953b6bb27257fa695a95d2f | 0.008 | PARL | 0.15 | *Firmicutes* | *Ruminococcaceae* | *Saccharofermentans* |
| ASV_73 | 74645290d255804647977d7a7006e5d1 | 0.008 | PARL | 0.01 | *Firmicutes* | *Lachnospiraceae* | [*Eubacterium*] hallii group |
| ASV_74 | 8e072eb8cd242c8ad3cc87d073adda61 | 0.008 | PARL | 0.02 | *Tenericutes* | *Anaeroplasmataceae* | *Anaeroplasma* |
| ASV_45 | 59ba4f126c09944012a362ae7f110e3d | 0.008 | PARL | 0.11 | *Firmicutes* | *Ruminococcaceae* | *Ruminococcus* 1 |
| ASV_75 | dc30740926a6165f607b5344c18b414c | 0.008 | PARL | 0.02 | *Firmicutes* | *Lachnospiraceae* | *Butyrivibrio* 2 |
| ASV_76 | 3c5a4988ec3713f16b12e779b63f4a85 | 0.007 | PARL | 0.01 | *Firmicutes* | *Lachnospiraceae* | *Lachnospiraceae* ND3007 group |
| ASV_10 | a9e241c1ad23939c707499e2eb74d04c | 0.007 | PARL | 0.14 | *Firmicutes* | *Lachnospiraceae* | *Lachnospiraceae* NK3A20 group |
| ASV_77 | 0dc9b00f2a52fddd3b84b16b80483892 | 0.007 | PARL | 0.02 | *Firmicutes* | *Lachnospiraceae* | *Lachnospiraceae* NK4A136 group |
| ASV_78 | 5ebd9238c9de787e60a2eaaa680ea049 | 0.007 | PARL | 0.11 | *Firmicutes* | *Lachnospiraceae* | *Acetitomaculum* |
| ASV_79 | af1306d2b695acfd05e69aa187d73a12 | 0.007 | PARL | 0.04 | *Firmicutes* | *Lactobacillaceae* | *Pediococcus* |
| ASV_80 | 73423c6c2f5f0258f4feb9d8fabe2836 | 0.007 | PARL | 0.01 | *Firmicutes* | *Ruminococcaceae* | *Ruminococcaceae* UCG-014 |
| ASV_81 | 631b177705ddcab7106be818a6a52db6 | 0.007 | PARL | 0.08 | *Firmicutes* | *Lachnospiraceae* | *Acetitomaculum* |
| ASV_82 | 479383ae1fe7dc01dbe0b340f3f25a61 | 0.007 | PARL | 0.03 | *Spirochaetes* | uncultured rumen bacterium | uncultured rumen bacterium |
| ASV_83 | d5184090af41259eac7d3eb7c79d7ec5 | 0.007 | PARL | 0.02 | *Actinobacteria* | *Atopobiaceae* | *Olsenella* |
| ASV_84 | 7ded4232bf1982b6061a233a7e75c014 | 0.007 | PARL | 0.04 | *Firmicutes* | *Lachnospiraceae* | [*Eubacterium*] xylanophilum group |
| ASV_85 | 5cf71c6f8524818fd9c8774dae34fd62 | 0.007 | PARL | 0.03 | *Firmicutes* | *Ruminococcaceae* | *Papillibacter* |
| ASV_86 | 17a6dd55d069685f74a04def313333cd | 0.006 | PARL | 0.01 | *Bacteroidetes* | *Prevotellaceae* | *Prevotellaceae* UCG-001 |
| ASV_87 | 229da9ff9ead4264b057a828870df3b3 | 0.006 | PARL | 0.02 | *Firmicutes* | *Lactobacillaceae* | *Lactobacillus* |
| ASV_88 | 88d12d817e72b6844f8dd87082299b23 | 0.005 | PARL | 0.01 | *Firmicutes* | *Christensenellaceae* | *Christensenellaceae* R-7 group |
| ASV_89 | 0af9c6d177f3ab0cb0ebbd756ba460ab | 0.005 | PARL | 0.05 | *Firmicutes* | *Lachnospiraceae* |  |
| ASV_90 | 84eff40552b5a31cd2e45aca11b8d3c7 | 0.005 | PARL | 0.28 | *Firmicutes* | *Ruminococcaceae* | *Ruminococcaceae* NK4A214 group |
| ASV_91 | 26337612d07d0270ef5662538fc46bdf | 0.004 | PARL | 0.03 | *Firmicutes* | *Ruminococcaceae* | *Ruminococcus* 1 |
| ASV_92 | 427b13524f5fe61773e96a03a7d84b22 | 0.003 | PARL | 0.10 | *Firmicutes* | *Lachnospiraceae* | *Lachnospiraceae* AC2044 group |
| ASV_93 | 2a4412d9babd4f4c59552b471eb66353 | 0.003 | PARL | 0.06 | *Firmicutes* | *Lachnospiraceae* | uncultured |
| ASV_94 | f784f809f7421be471ac0c2f6e6d8fdb | 0.003 | PARL | 0.07 | *Firmicutes* | *Ruminococcaceae* | *Ruminococcaceae* UCG-014 |
| ASV_95 | 60fe24ba7378d81be8f1e8a341ddf5ab | 0.003 | PARL | 0.52 | *Firmicutes* | *Lachnospiraceae* | *Lachnospiraceae* NK3A20 group |
| ASV_96 | 6c1cb6d9bdf0db46bc079a46703f9a77 | 0.003 | PARL | 0.01 | *Firmicutes* | *Ruminococcaceae* | *Ruminococcaceae* UCG-005 |
| ASV_97 | a521bf3a12b065bb39d64b7ca669787c | 0.125 | Feces | 0.04 | *Firmicutes* | *Lachnospiraceae* | *Lachnospiraceae* NK3A20 group |
| ASV_98 | 953d73633263164d4c1f46373cb2022c | 0.064 | Feces | 0.18 | *Firmicutes* | *Lachnospiraceae* | *Lachnospiraceae* NK3A20 group |
| ASV_99 | b02086cd3059a5b286dc3d5995a4cb6f | 0.049 | Feces | 0.04 | *Firmicutes* | *Lachnospiraceae* | *Marvinbryantia* |
| ASV_100 | a4303649a57e8a6efe59ba2324e7d499 | 0.049 | Feces | 0.23 | *Firmicutes* | Family XIII | Family XIII AD3011 group |
| ASV_101 | 12d3525fada9318ff868c6d24d1f05e5 | 0.046 | Feces | 0.65 | *Firmicutes* | *Ruminococcaceae* | *Ruminococcaceae* UCG-005 |
| ASV_102 | a5f1e82821c17bf542f5598cc9d3708e | 0.043 | Feces | 0.22 | *Firmicutes* | Family XIII | Family XIII AD3011 group |
| ASV_103 | 644f478e702864f3b0b0cf22ab471746 | 0.036 | Feces | 0.21 | *Firmicutes* | *Ruminococcaceae* | *Ruminococcaceae* UCG-005 |
| ASV_104 | 016513c1b6447445eb4fd91fd7e3ff3a | 0.032 | Feces | 0.08 | *Firmicutes* | *Lachnospiraceae* | *Anaerosporobacter* |
| ASV_105 | 4d628db4c55bdf2b9e685612d282b7fe | 0.028 | Feces | 0.05 | *Firmicutes* | *Ruminococcaceae* | *Negativibacillus* |
| ASV_106 | fb30e43af9ecc53d354d0e3b98a42287 | 0.020 | Feces | 0.04 | *Firmicutes* | *Lachnospiraceae* |  |
| ASV_107 | f0cea68ddfe1201a78348dc2e97d6c7d | 0.016 | Feces | 0.11 | *Firmicutes* | *Lachnospiraceae* | *Blautia* |
| ASV_108 | b5477f82c4b13260d52a51057e0f41e9 | 0.014 | Feces | 0.03 | *Firmicutes* | *Lachnospiraceae* | *Anaerosporobacter* |
| ASV_109 | 95e6ec27d9f89aaf420792f42f599146 | 0.014 | Feces | 0.21 | *Firmicutes* | *Lachnospiraceae* | *Lachnospiraceae* FE2018 group |
| ASV_110 | 5da6982a564fb7b0e185189ac00d706a | 0.014 | Feces | 0.02 | *Firmicutes* | *Ruminococcaceae* | *Ruminococcaceae* UCG-010 |
| ASV_111 | 2d6fd3e2ce49e992a17d8696a705e005 | 0.013 | Feces | 0.18 | *Firmicutes* | *Ruminococcaceae* | *Ruminococcaceae* UCG-009 |
| ASV_112 | 0d665cc3a5efb3379a3b4e73aa6813fa | 0.012 | Feces | 0.80 | *Bacteroidetes* | *Bacteroidales* RF16 group | uncultured *Parabacteroides* sp. |
| ASV_113 | 42e0917a2cb94fe636a0b0e0f8746894 | 0.008 | Feces | 0.15 | *Firmicutes* | *Ruminococcaceae* | *Ruminococcaceae* UCG-010 |
| ASV_114 | 5d3b14e48a840f6d5fa4bcfdb9d69f63 | 0.008 | Feces | 0.08 | *Patescibacteria* | *Saccharimonadaceae* | *Candidatus* *Saccharimonas* |
| ASV_115 | b7cf7afd33da98a0c06092954892a1fe | 0.008 | Feces | 0.03 | *Firmicutes* | *Lachnospiraceae* | *Lachnospiraceae* NK3A20 group |
| ASV_116 | 1612ac89a302eeba2ffb3190be417595 | 0.008 | Feces | 0.23 | *Firmicutes* | *Lachnospiraceae* | [*Eubacterium*] hallii group |
| ASV_117 | e98a8b47af77547e0b117d7ed5f54383 | 0.007 | Feces | 0.17 | *Bacteroidetes* | *Bacteroidaceae* | *Bacteroides* |
| ASV_118 | efadd5f0dcc9ceffdb2da68a3d9f24fa | 0.007 | Feces | 0.06 | *Firmicutes* | *Ruminococcaceae* |  |
| ASV_119 | d951bb250962046dfa6d2c1e7f275e70 | 0.007 | Feces | 0.02 | *Firmicutes* | *Lachnospiraceae* | *Blautia* |
| ASV_120 | 17ea365d039598c8fbebaebcfde1f4df | 0.007 | Feces | 0.06 | *Firmicutes* | *Christensenellaceae* | *Christensenellaceae* R-7 group |
| ASV_121 | b9256420e9da081422523b7d22c6f1f3 | 0.007 | Feces | 0.01 | *Firmicutes* | *Ruminococcaceae* | *Ruminococcaceae* UCG-014 |
| ASV_122 | be75533d5a75eaac7876f20c2a6c74ba | 0.007 | Feces | 1.16 | *Firmicutes* | *Lachnospiraceae* | *Lachnospiraceae* AC2044 group |
| ASV_123 | 5e05470f737b3e65161f43663e601f95 | 0.007 | Feces | 0.02 | *Firmicutes* | *Ruminococcaceae* | *Ruminococcaceae* UCG-010 |
| ASV_124 | 2ec4fbec01b38dcccfe19a5c9c273557 | 0.007 | Feces | 0.21 | *Firmicutes* | *Lachnospiraceae* | *Marvinbryantia* |
| ASV_125 | 65603b150f85a5676710e99d0fc1b561 | 0.007 | Feces | 0.24 | *Verrucomicrobia* | *Akkermansiaceae* | *Akkermansia* |
| ASV_126 | 666b3c14006e6ded0b85d4f19aaf5bf5 | 0.007 | Feces | 0.12 | *Firmicutes* | *Lachnospiraceae* | *Lachnospiraceae* NK4A136 group |
| ASV_127 | 5437fcf4d3e4a12d2b23b582d98b6bee | 0.007 | Feces | 0.09 | *Bacteroidetes* | *Rikenellaceae* | *Rikenellaceae* RC9 gut group |
| ASV_128 | 245becfa2efaf0d7e93579b78fc82684 | 0.006 | Feces | 0.11 | *Firmicutes* | Family XIII | Family XIII AD3011 group |
| ASV_129 | bd88d67a4c7d090c24a9ff33d9fb7a2b | 0.006 | Feces | 0.03 | *Lentisphaerae* | *Victivallaceae* | uncultured bacterium |
| ASV_130 | 5d5bbac5b2746b0062927dd2661c9352 | 0.006 | Feces | 0.02 | *Firmicutes* | *Ruminococcaceae* | *Ruminococcaceae* UCG-010 |
| ASV_131 | 022f1ef4f51d7f49923c4cdaf137d842 | 0.006 | Feces | 0.03 | *Firmicutes* | *Ruminococcaceae* | [*Eubacterium*] coprostanoligenes group |
| ASV_132 | 1f9c17dec8f01bd67683280c221fe523 | 0.006 | Feces | 0.02 | *Firmicutes* | *Lachnospiraceae* | *Lachnospiraceae* NK3A20 group |
| ASV_133 | f7f3103ef2627f1849ffb8e11ba5fbd3 | 0.006 | Feces | 0.02 | *Firmicutes* | *Lachnospiraceae* | *Lachnospiraceae* NK3A20 group |
| ASV_134 | 47067be3005daa78970a472ea9ce2306 | 0.006 | Feces | 0.08 | *Firmicutes* | *Ruminococcaceae* | *Ruminococcaceae* UCG-013 |
| ASV_135 | fd6d9bb2dfc71ca76ee04783ab9bd3ee | 0.006 | Feces | 0.07 | *Firmicutes* | *Ruminococcaceae* | [*Eubacterium*] coprostanoligenes group |
| ASV_136 | 05ac956b3264d95d2da4aff16e2c68c9 | 0.005 | Feces | 0.16 | *Firmicutes* | *Ruminococcaceae* | *Ruminococcaceae* UCG-010 |
| ASV_137 | 231969e6bfbf1a19b2a07e4aa130c55d | 0.005 | Feces | 0.03 | *Firmicutes* | *Christensenellaceae* | *Christensenellaceae* R-7 group |
| ASV_138 | 4d3d55a0bd2aa2c717f7f4d82a71aa05 | 0.005 | Feces | 0.04 | *Firmicutes* | *Ruminococcaceae* | [*Eubacterium*] coprostanoligenes group |
| ASV_139 | 9292f3f14ba9bd19279d5bff4e288fa3 | 0.005 | Feces | 0.38 | *Firmicutes* | *Ruminococcaceae* | *Ruminococcaceae* UCG-005 |
| ASV_140 | c654c72f3d69cb8952aa5f298003dff4 | 0.004 | Feces | 0.01 | *Firmicutes* | *Christensenellaceae* | *Christensenellaceae* R-7 group |
| ASV_141 | bf1c82ff600cd9ff1a9b369c1e3a95f1 | 0.004 | Feces | 0.03 | *Bacteroidetes* | *Rikenellaceae* | *Rikenellaceae* RC9 gut group |
| ASV_142 | c150a4ae4e5473c225e82e9bf77e3bbf | 0.004 | Feces | 0.04 | *Firmicutes* | *Lachnospiraceae* | *Lachnospiraceae* NK3A20 group |
| ASV_143 | d980b18fd704eaf3d2f46f3fe90a4636 | 0.004 | Feces | 0.05 | *Firmicutes* | *Lachnospiraceae* |  |
| ASV_144 | 50e5bacf4d693c48094f83c4702de668 | 0.004 | Feces | 0.07 | *Firmicutes* | *Lachnospiraceae* | *Cellulosilyticum* |
| ASV_145 | 6c5c58c160beed1e572e84df5c32e47f | 0.003 | Feces | 0.08 | *Bacteroidetes* | *Prevotellaceae* | *Prevotellaceae* UCG-003 |
| ASV_146 | a07c965063a39f1c87025dacc4bf2e9b | 0.003 | Feces | 0.36 | *Firmicutes* | *Ruminococcaceae* | [*Eubacterium*] coprostanoligenes group |

##
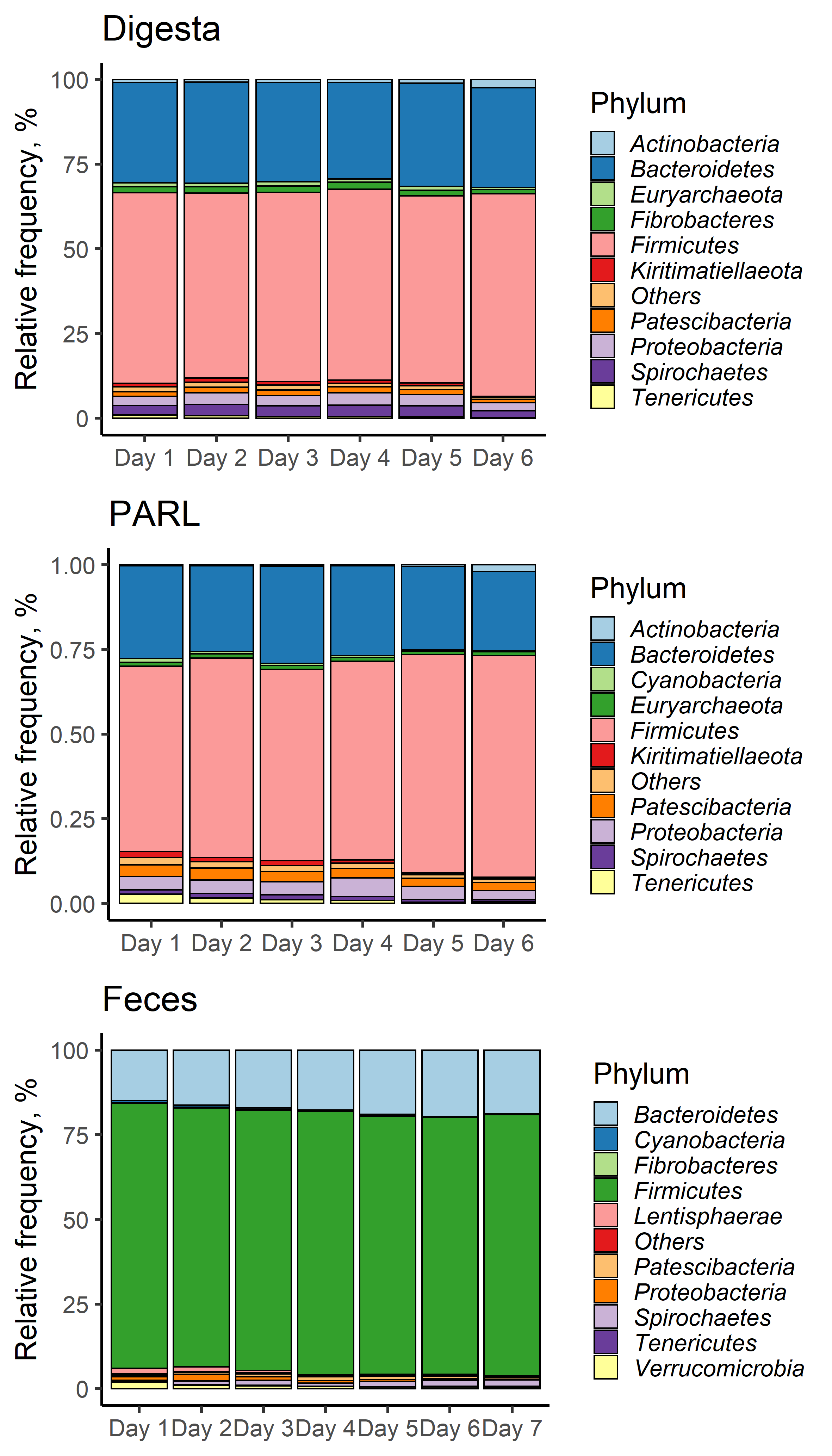
Supplementary Figures

**Supplementary Figure S1.** Mean relative frequency of the 10 most abundant phyla (across all samples) detected in the three analyzed niches. Results are presented for solid digesta (Digesta), particle associated rumen liquid (PARL) and fecal samples over the experimental days. Rumen samples were collected for 6 days, while feces were sampled for an additional seventh day.


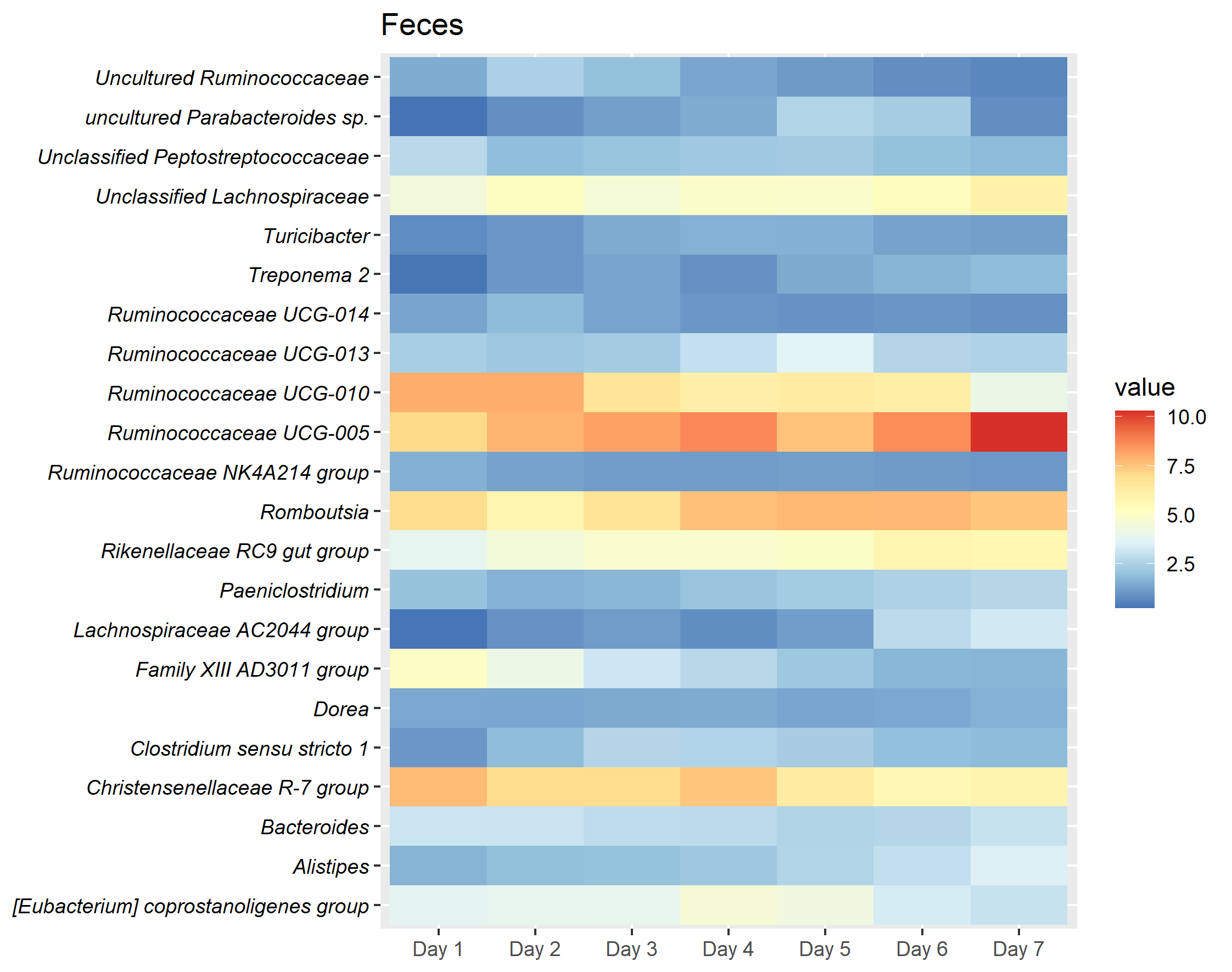


**Supplementary Figure** **S2.** Heatmap showing the microbial composition of the fecal samples. The mean relative frequency of the 25 most abundant genera across all samples is presented for each experimental day.


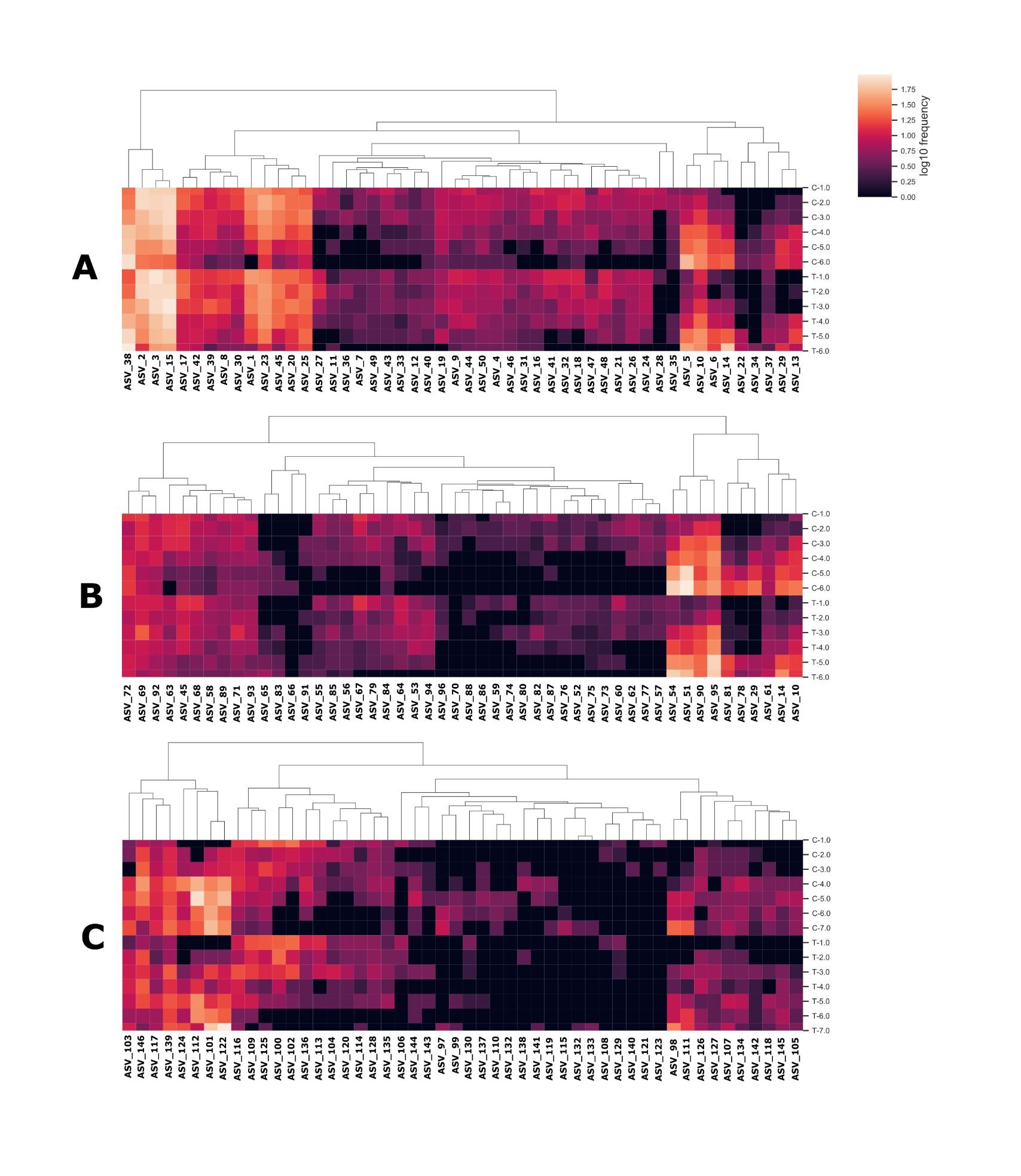


**Supplementary Figure S3.** Heatmaps showing the frequency of the 50 most important features detected via random forest regression. The taxa were detected in QIIME2 via “longitudinal maturity-index”, performing a random forest regression for solid digesta (**A**), particle associated rumen liquid (PARL) (**B**) and feces (**C**). Individual samples are merged per each sampling day (1 to 6 and 1 to 7 for feces) for control (C) and treatment (T) groups. ASVs details and taxonomy are given in Table S7.


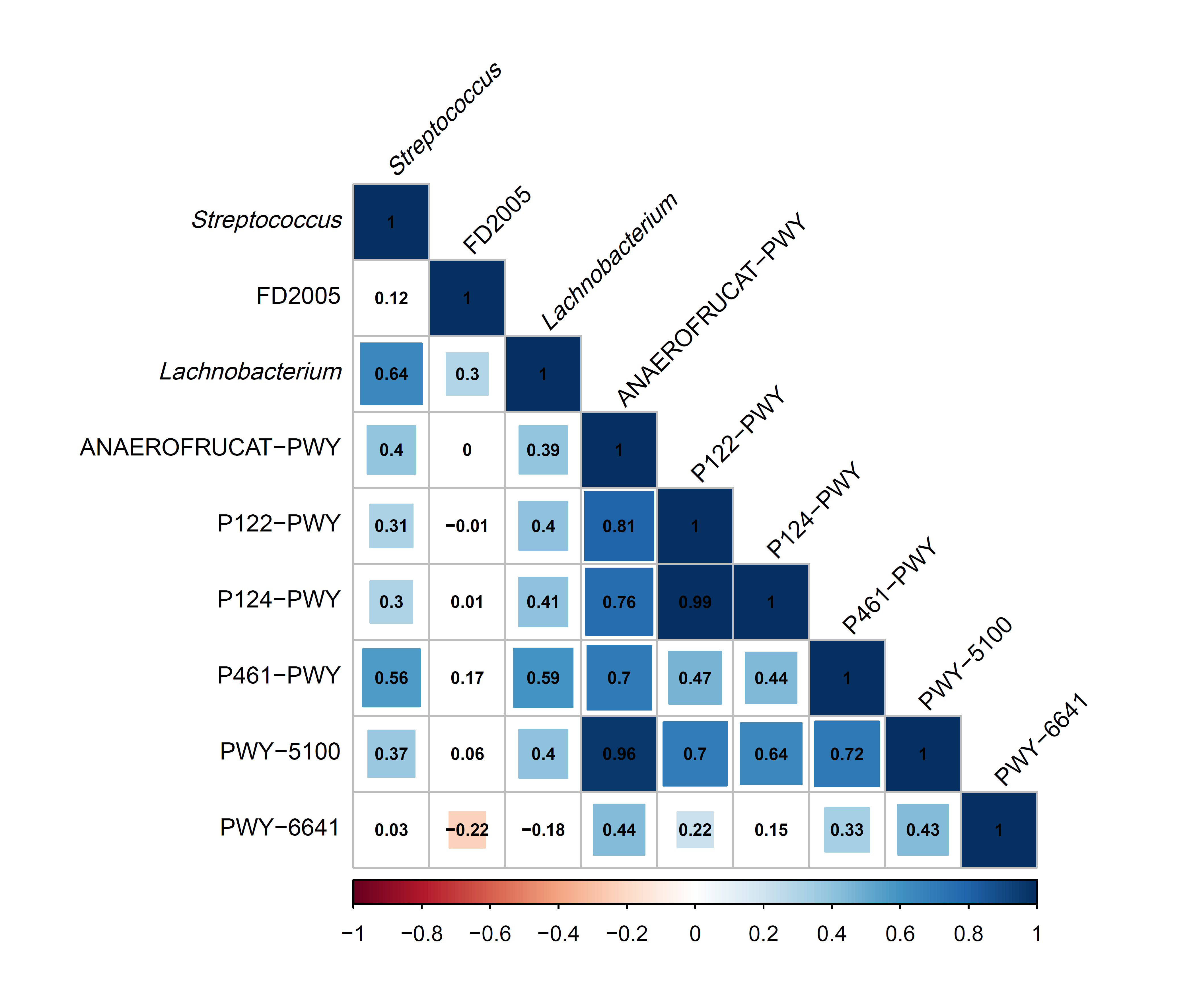


**Supplementary Figure S4.** Correlogram showing Spearman correlations between taxa and predicted pathways associated with lactate metabolism. The same taxa were found to be positively correlated with D-lactate concentration measured in the rumen. Correlations were calculated using R package Hmisc while the correlogram was produced using corrplot package.
